# Dual Targeting of DNA and EGFR by ZYH005 Induces DNA Damage and Mitotic Catastrophe in Glioblastoma

**DOI:** 10.1002/mco2.70717

**Published:** 2026-04-01

**Authors:** Jianzheng Huang, Zijun Zhang, Yang Xiao, Ziming Zhao, Zengwei Luo, Junjun Liu, Suitian Lai, Chao Song, Shouchang Feng, Suojun Zhang, Xingjiang Yu, Qingyi Tong, Yonghui Zhang

**Affiliations:** ^1^ Department of Hubei Key Laboratory of Natural Medicinal Chemistry and Resource Evaluation School of Pharmacy Tongji Medical College Huazhong University of Science and Technology Wuhan China; ^2^ Department of Histology and Embryology School of Basic Medicine Tongji Medical College Huazhong University of Science and Technology Wuhan China; ^3^ Department of Neurosurgery Tongji Hospital Tongji Medical College Huazhong University of Science and Technology Wuhan China

**Keywords:** DNA damage, epidermal growth factor receptor–WEE1 axis, glioblastoma, mitotic catastrophe

## Abstract

Glioblastoma multiforme (GBM) is an aggressive, therapy‐resistant brain tumor with limited treatment options. Epidermal growth factor receptor (EGFR) drives GBM pathogenesis. Here, we investigate ZYH005 (Z5), a brain‐penetrant DNA intercalator with low systemic toxicity, as a novel therapeutic agent. Z5 potently inhibits the proliferation of GBM cell lines and patient‐derived glioblastoma stem cells (GSCs) in vitro and suppresses tumor growth in orthotopic GSCs‐derived mouse models, significantly prolonging survival without apparent toxicity. Mechanistically, Z5 exerts potent anti‐GBM activity through a dual mechanism: DNA intercalation‐induced damage and targeted inhibition of EGFR. By specifically inhibiting EGFR at E762, Z5 not only enhances DNA damage by suppressing the DNA damage response in the nucleus but also disrupts the interaction between nuclear EGFR and WEE1, leading to impaired WEE1/CDC2 signaling and G2/M checkpoint failure. Extranuclearly, Z5 further enhances its anti‐GBM efficacy by inhibiting the canonical EGFR downstream pathways, mTOR, and ERK. These combined actions lead to cell cycle arrest and mitotic catastrophe. Our findings establish Z5 as a promising clinical candidate for classical GBM, employing a unique dual mechanism that overcomes EGFR‐targeted and DNA‐damaging therapy limitations by synergistically targeting DNA and EGFR with high efficacy, advancing understanding of EGFR–WEE1 biology, and supporting clinical development.

## Graphical abstract

ZYH005 exerts potent anti‐glioblastoma activity through a dual mechanism: intercalation‐mediated DNA damage and targeted inhibition of EGFR, leading to cell cycle arrest and mitotic catastrophe.

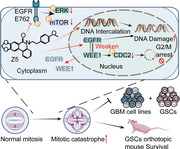

## 1 Introduction

Glioblastoma multiforme (GBM) stands as the most invasive and deadly primary brain tumor among adults [1]. It constitutes around 49% of all malignant central nervous system tumors. In high‐income countries, its annual incidence is 3–7 cases per 100,000 individuals [2, 3]. The treatment of GBM adopts a multimodal strategy, integrating surgery, radiotherapy, and temozolomide (TMZ)‐based chemotherapy. However, the median survival time for GBM patients is a mere 12–16 months, and the 5‐year survival rate remains below 5% [2, 3]. This persistent therapeutic impasse underscores the critical need for paradigm‐shifting treatment strategies in neuro‐oncology.

Understanding the molecular heterogeneity of GBM is therefore essential for developing targeted therapies. Transcriptomic profiling has classified GBM into three major molecular subtypes—classical (CL), proneural (PN), and mesenchymal (MES)—which differ in genetic alterations and clinical behavior [4]. Notably, the classical subtype is predominantly defined by aberrations in the epidermal growth factor receptor (EGFR) gene [5], whereas proneural tumors frequently harbor PDGFRA alterations and IDH1 mutations, and mesenchymal tumors are enriched for NF1 loss and inflammatory signatures [6]. Across all subtypes, EGFR amplification and mutation occur in more than half of GBM cases, establishing EGFR as a central oncogenic driver. Beyond activating canonical signaling pathways such as PI3K/AKT/mTOR, RAS/MAPK, and JAK/STAT—which collectively promote tumor proliferation, survival, and invasion—EGFR also translocates to the nucleus, where it directly facilitates DNA repair. There, it interacts with DNA‐PKcs to enhance its phosphorylation, bolstering nonhomologous end joining (NHEJ) [7, 8]. Furthermore, through the RAS/RAF/MEK/ERK and PI3K/AKT cascades, EGFR modulates the expression of DNA repair factors such as XRCC1 and RAD51, thereby enhancing DNA damage response (DDR) homeostasis and aiding tumor cells in evading genotoxic stress [9, 10]. This central role of EGFR in coordinating DDR underlies why its pharmacological inhibition not only disrupts DNA repair mechanisms but also sensitizes GBM cells to radiotherapy and DNA‐damaging agents [11, 12]. Together, these multifaceted functions solidify EGFR's position as a cornerstone therapeutic target in GBM.

Tyrosine kinase inhibitors (TKIs) show inhibitory activity against EGFR; their monotherapy efficacy in GBM is limited due to tumor heterogeneity, compensatory signaling pathways, and the blood–brain barrier (BBB) [13]. Accordingly, combination strategies—particularly pairing EGFR–TKIs with MEK/PI3K inhibitors or DNA‐damaging agents (e.g., temozolomide, radiotherapy)—are required to achieve superior therapeutic outcomes. Preclinical studies further demonstrate that timed EGFR–TKI administration (≥4 h before DNA damage induction) potentiates tumor cell killing by prolonging DDR pathway disruption [14, 15]. However, combination regimens are often limited by increased toxicity, complex pharmacokinetics, and challenges in optimizing drug sequencing and dosing. These limitations have spurred growing interest in dual‐targeting strategies that simultaneously disrupt EGFR signaling and induce DNA damage. A representative example is ZR2002, a small‐molecule agent designed to covalently modify DNA bases via a haloalkyl arm, thereby compromising DNA integrity while concurrently inhibiting EGFR activity. ZR2002 exhibits potent submicromolar antiproliferative activity even against temozolomide‐resistant GSCs [16], highlighting the promise of co‐targeting EGFR and DNA.

Phenanthridinone represents an important class of heterocyclic frameworks frequently found in bioactive alkaloids [17] and serves as a core structure with significant potential in antitumor drug discovery [18, 19]. Previously, we synthesized a series of novel phenanthridinone derivatives and identified a crinasiadine‐type derivative, ZYH005 (Z5), as the most potent compound, exhibiting strong anti‐acute myeloid leukemia (AML) activity [19]. Mechanistically, Z5 exerts its anti‐AML effects by intercalating into DNA to induce DNA damage and triggering the caspase‐dependent degradation of PML‐RARα, ultimately leading to AML cell apoptosis. While Z5 shows great promise, its potential for treating GBM remains unexplored. Insights from related phenanthridinone alkaloids suggest a viable path forward: narciclasine has demonstrated significant inhibition of primary GBM and brain‐metastatic cancers in mouse models, attributed to its high lipophilicity and ability to penetrate the BBB [20, 21, 22]. Similarly, lycorine induces apoptosis in GBM cells and patient‐derived xenograft models via EGFR inhibition [23], and narciclasine suppresses gastric cancer proliferation by modulating the AKT/mTOR pathway linked to EGFR signaling [24]. These findings underscore the therapeutic potential of phenanthridinones targeting EGFR‐related mechanisms. Structurally, Z5 possesses fewer hydrophilic hydroxyl groups than narciclasine, suggesting higher theoretical lipophilicity. Given its superior structural attributes and proven potency, it is therefore crucial to investigate whether Z5 possesses anti‐GBM efficacy and to elucidate its underlying molecular mechanisms.

In the present study, we clearly demonstrated that Z5 exerts a significant inhibitory effect on GBM cells and patient‐derived GSCs in vitro and in vivo. We confirmed for the first time that Z5 exerts anti‐GBM effects through a unique dual‐mechanism approach, acting as a DNA intercalator and a selective EGFR inhibitor. These findings not only provide a mechanistic foundation for the development of Z5 as a lead compound but also highlight a novel therapeutic hope for GBM, demonstrating compelling potential against this aggressive malignancy and warranting further investigation.

## 2 Results

### 2.1 Z5 suppresses Proliferation and Inhibits EGFR‐Mediated Signaling in GBM Cell Lines

**FIGURE 1:**
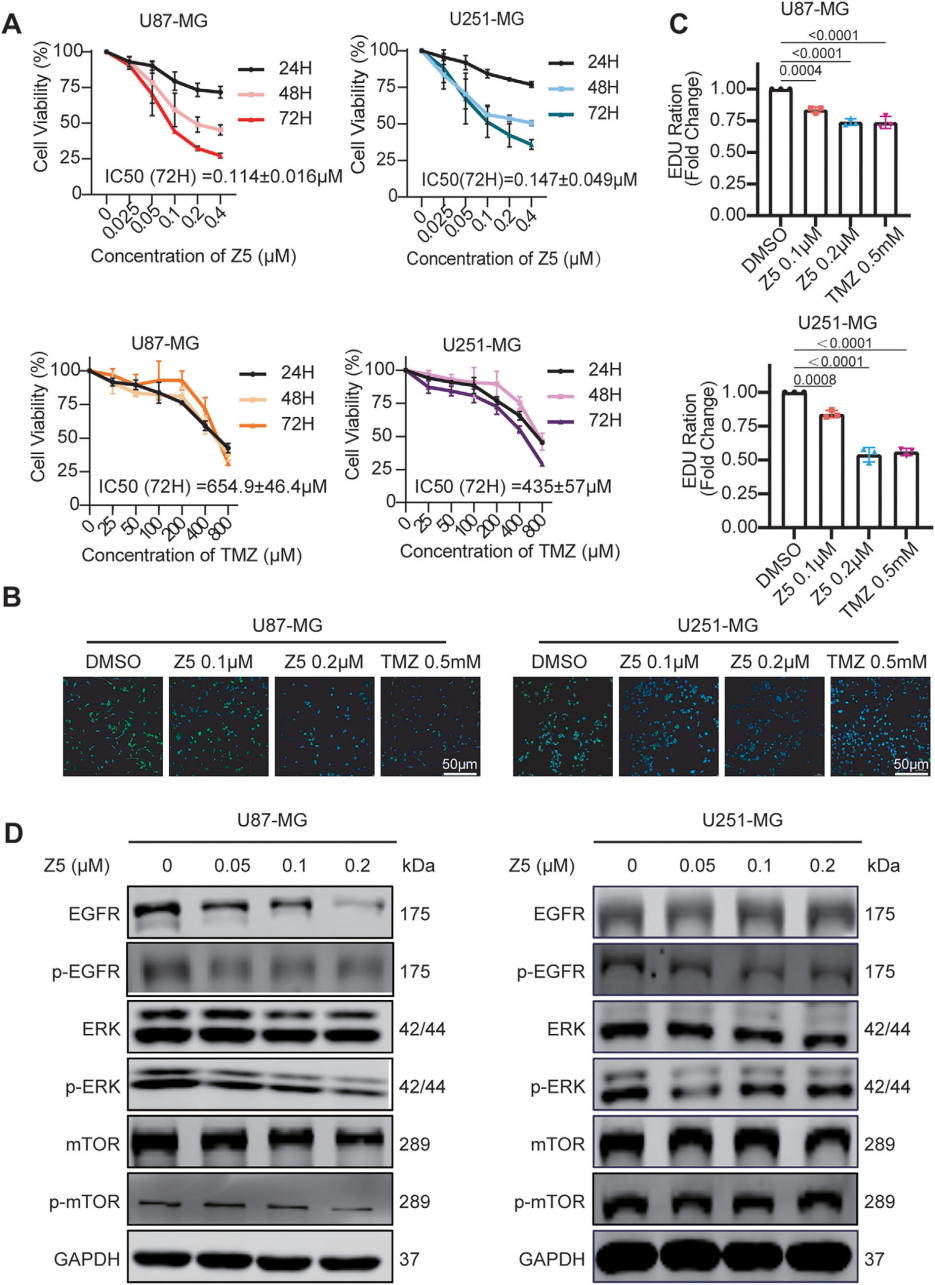
Z5 suppresses proliferation and blocks EGFR‐mediated signaling in GBM cell lines. (A) Cell viability of GBM cell lines was assessed by CCK‐8 assays after treatment with Z5 or TMZ for 24, 48, and 72 h. *n* = 3 (B)Fluorescence images showing EdU incorporation in GBM cells under various treatment conditions (green: EdU staining; blue: Hoechst staining). (C) Quantification of EdU‐positive cells in each group. The data are presented as the mean ± SD; *n* = 3, compared with the DMSO group. (D) The expression levels of EGFR, p‐EGFR, ERK, p‐ERK, mTOR, and p‐mTOR were analyzed by immunoblotting after Z5 treatment.

Z5, a narciclasine analog with reduced hydrophilicity (Figure S1A), demonstrated efficient blood—brain barrier penetration in mice, as evidenced by sustained brain exposure and brain‐to‐plasma ratios exceeding 0.3 over 2 h (Figure S1B–D). We therefore evaluated its antitumor activity in glioblastoma models. Using two representative GBM cell lines, U87‐MG (Mesenchymal type [25]) and U251‐MG (Classical type), we demonstrated that Z5 potently inhibits GBM cell proliferation in a time‐ and concentration‐dependent manner (Figure S2A). After 72 h of treatment, the IC_50_ values were 0.114 ± 0.016 µM for U87‑MG and 0.147 ± 0.049 µM for U251‑MG, whereas those of temozolomide (TMZ) were 654.9 ± 46.4 µM and 435 ± 57 µM, respectively (Figure 1A). Notably, treatment with 0.2 µM Z5 for 48 h resulted in approximately 50% inhibition of cell proliferation in both cell lines, whereas the IC_50_ value of lycorine in U251‐MG cells was 10 µM under the same conditions [23], indicating that Z5 exhibits superior antiproliferative activity to lycorine in GBM cells.

Furthermore, Z5 significantly suppressed colony formation in both cell lines (Figure S3A,B). The EdU incorporation assay further demonstrated marked inhibition of DNA synthesis following Z5 treatment, a key characteristic of suppressed cell proliferation (Figure 1B,C). Consistent with its antiproliferative effects and similar to the activity of lycorine [23], treatment with Z5 also significantly suppressed the EGFR signaling pathway, as demonstrated by reduced phosphorylation levels of key biomarkers including EGFR (p‐EGFR), ERK (p‐ERK), and mTOR (p‐mTOR) (Figure 1D).

### 2.2 Z5 suppresses GBM Partly Through Selective Targeting of EGFR

**FIGURE 2:**
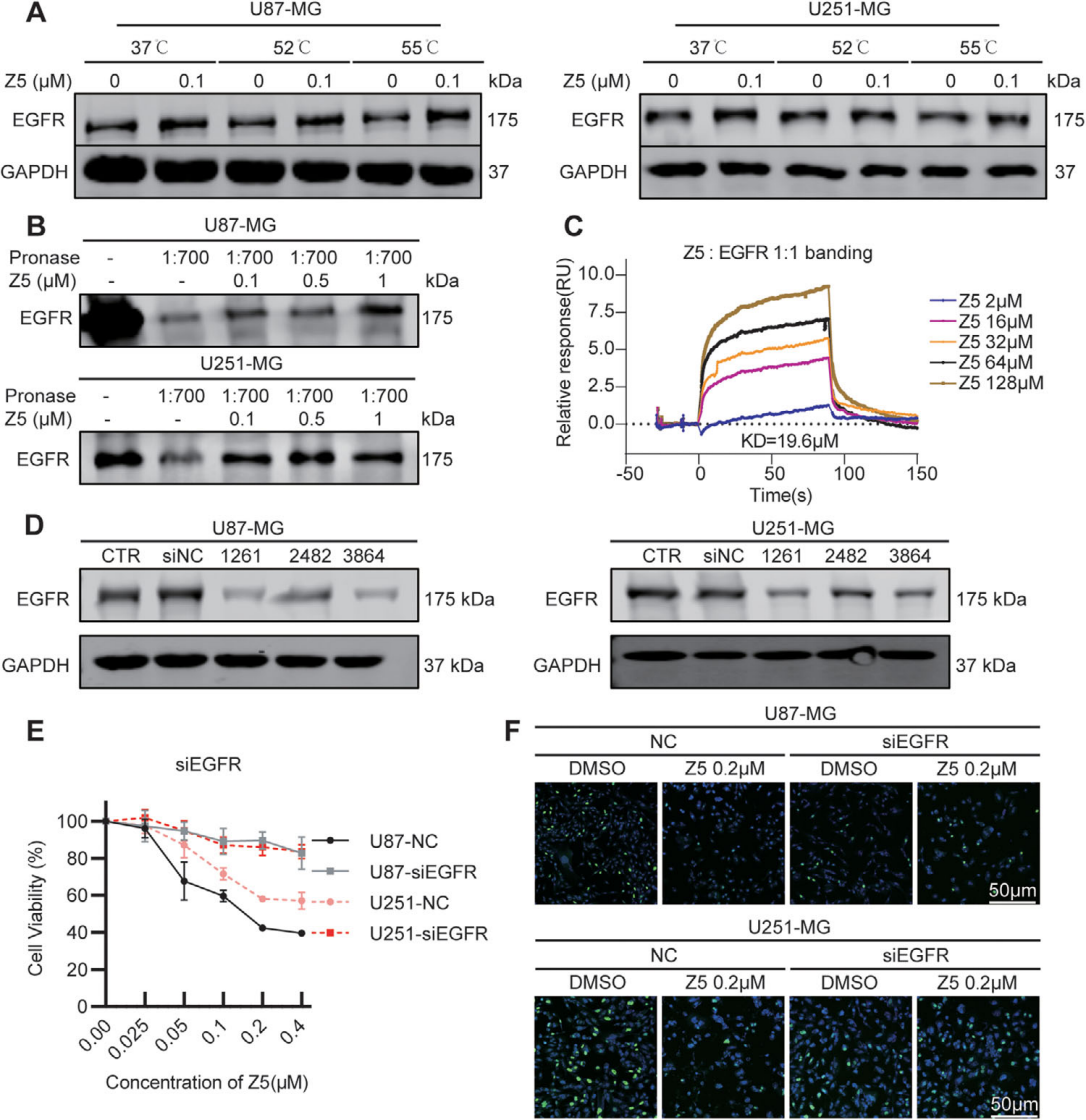
Z5 suppresses GBM partly through selective targeting of EGFR. (A) CETSA assay was performed to assess target engagement of Z5 (0.1 mM) with EGFR in cells. EGFR expression was detected by immunoblotting. (B) The DARTS assay was used to evaluate the direct binding of Z5 to EGFR at varying concentrations. EGFR expression was detected by immunoblotting. (C) The direct binding affinity between Z5 and the recombinant EGFR kinase domain protein was analyzed by SPR. (D) EGFR silencing efficiency was evaluated by immunoblot analysis after transfection with EGFR siRNA. (E) Growth curves of NC and si‐EGFR‐1261 GBM cell lines were measured following treatment with Z5 for 48 h. *n* = 3. (F) The antiproliferative effect of Z5 in NC and si‐EGFR‐1261 GBM cells was assessed by EdU assay.

To determine whether Z5 directly targets EGFR, we performed a series of biochemical assays. Cellular thermal shift assay (CETSA) demonstrated that Z5 enhanced the thermal stability of EGFR in both U87‐MG and U251‐MG cell lines (Figure 2A), whereas drug affinity responsive target stability (DARTS) assay also revealed that Z5 protected EGFR from proteolytic degradation (Figure 2B). Most conclusively, surface plasmon resonance (SPR) experiments confirmed direct binding between Z5 and the EGFR kinase domain, with a dissociation constant (KD) of 19.6 µM (Figure 2C).

To confirm the interaction between Z5 and EGFR, we examined how EGFR silencing influences the antiproliferative effects induced by Z5. The knockdown efficiency of EGFR‐targeting siRNAs was first evaluated in GBM cell lines. Based on its superior performance, siEGFR‐1261 was selected for further experiments (Figure 2D). Our results demonstrate that EGFR knockdown attenuated—but did not abolish—the antiproliferative effect of Z5, as evidenced by CCK‐8 and EdU assays (Figure 2E,F). Together, these findings establish that Z5 targets EGFR and that its anti‐GBM efficacy is largely mediated through this interaction.

### 2.3 Z5 Binds to the E762 Residue of EGFR

**FIGURE 3:**
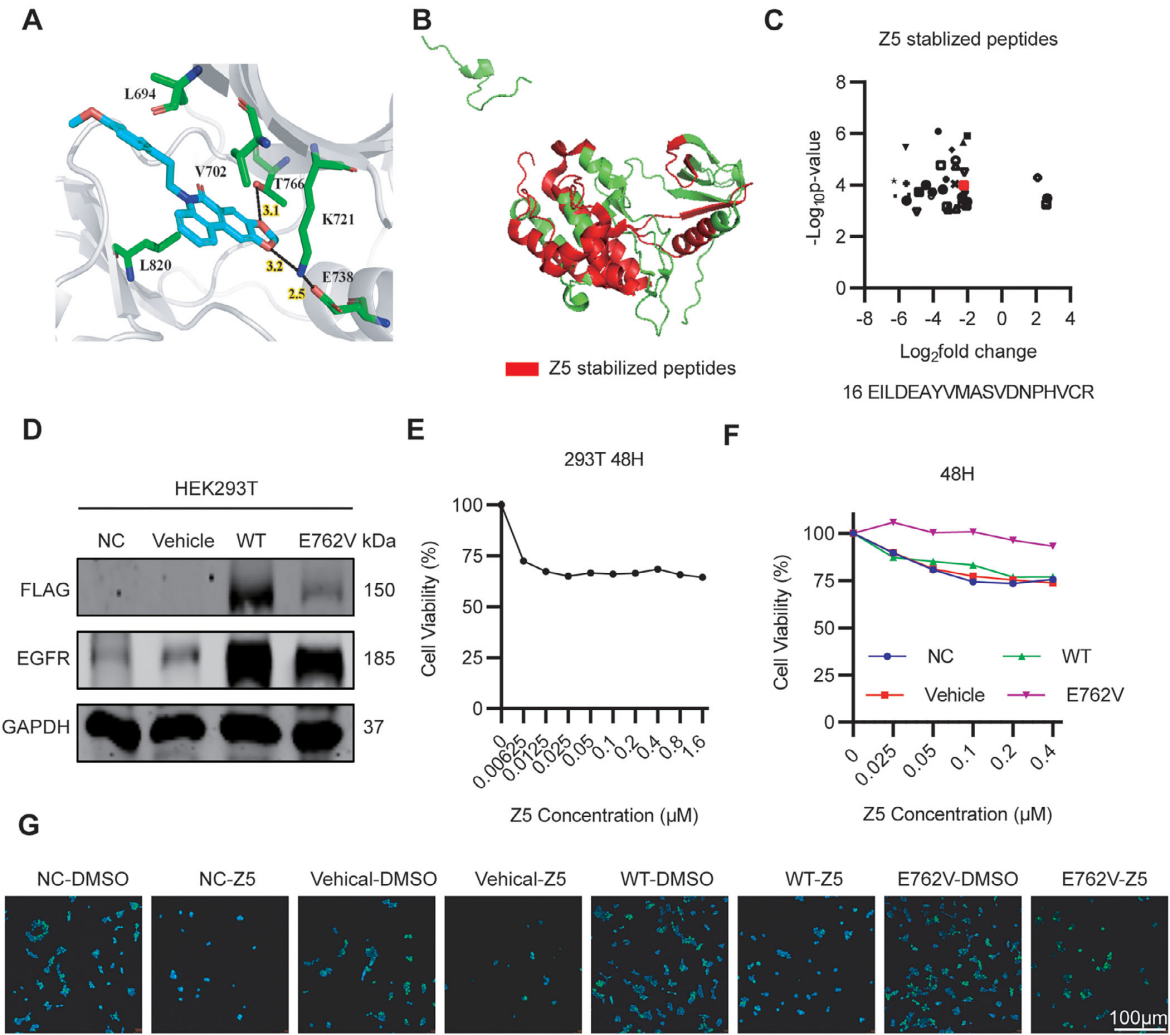
Z5 binds to the E762 residue of EGFR. (A) Predicted binding mode of Z5 within the EGFR kinase domain. (B) Red regions represent peptides with increased proteolytic susceptibility upon Z5 treatment. The selection criteria were as follows: log_2_(fold change) > 2 and −log_10_(P value) > 2. (C) Scatter plot displaying changes in peptide abundance from Lip‐MS analysis. Each point represents a peptide, with fold‐change differences shown between Z5‐treated and control samples. (D) Expression of Flag‐tagged EGFR and total EGFR in HEK293T cells was analyzed by immunoblotting after transfection with different plasmids. (E) Growth curves of HEK293T cells following treatment with Z5 for 48 h. (F) Growth curves of HEK293T cells transfected with empty vector (vehicle), wild‐type (WT) EGFR, or the E762V mutant EGFR, following treatment with Z5 for 48 h. (G) Fluorescence images of proliferating HEK293T cell lines under various treatment conditions (green: EdU staining; blue: Hoechst staining).

Having established that Z5 directly targets EGFR, we further investigated the structural basis of this interaction. Molecular docking simulations were performed targeting the ATP‐binding pocket of the EGFR kinase domain (PDB: 7KXZ). Detailed analysis of the binding mode revealed key molecular interactions between Z5 and EGFR: (1) extensive hydrophobic contacts with residues V702, L820, and L694, stabilizing its position within the ATP‐binding pocket; and (2) two critical hydrogen bonds formed with K721 and T766, which likely contribute to its high binding affinity (Figure 3A).

To experimentally validate these computational predictions, we employed limited proteolysis‒mass spectrometry (LiP‒MS), a label‐free proteomics approach, to map potential binding sites of Z5 within the EGFR kinase domain. The LiP‐MS analysis identified several peptide segments that are likely involved in Z5 binding (Figure 3B,C). Although the residues K721 and T766—previously implicated in hydrogen bonding by docking studies—were not directly detected by limited proteolysis–mass spectrometry (LiP–MS), we observed that K721 forms a hydrogen bond with E738. This interaction may stabilize K721 and thereby indirectly support its hydrogen bonding with Z5. Based on these integrated findings, we propose E738 (corresponding to E762 in UniProt entry P00533‐1) as a critical binding residue for Z5. To functionally validate the significance of E762, we established an ectopic expression system in HEK293T cells transfected with an empty vector, wild‐type (WT) EGFR, or the E762V mutant EGFR (Figure 3D). Dose‒response experiments using the CCK‐8 assay revealed that the antiproliferative effect of Z5 plateaued at concentrations above 0.1 µM, with residual cell viability stabilizing around 75% (Figure 3E). Based on these observations, we selected 0.2 µM Z5 for subsequent experiments to ensure detectable antiproliferative effects while avoiding complete cytotoxicity. Functional validation using both CCK‐8 and EdU assays demonstrated that the E762V mutation significantly compromised the antiproliferative effect of Z5 (Figure 3F,G). Compared with cells expressing WT‐EGFR, those carrying the E762V mutation exhibited markedly higher cell viability and enhanced proliferative capacity after Z5 treatment. These results confirm that E762 serves as a critical binding residue for Z5 on EGFR and is essential for mediating its antiproliferative activity.

### 2.4 Z5 Induces DNA Damage Through DNA Intercalation and EGFR Inhibition

To gain deeper insights into the molecular mechanisms underlying Z5‐mediated EGFR inhibition, we conducted a comprehensive microarray analysis in U87‐MG cells, which were selected due to their significantly greater sensitivity to Z5 observed in prior CCK‐8 and Western blot assays, thereby ensuring robust detection of transcriptional alterations. The analysis identified a total of 1970 differentially expressed genes (DEGs), among which 188 were upregulated, and 1782 were downregulated (Figure S4A). Notably, PRIM1, PRKDC, and MKI67 exhibited the most pronounced downregulation (Figure S4B,C).

**FIGURE 4:**
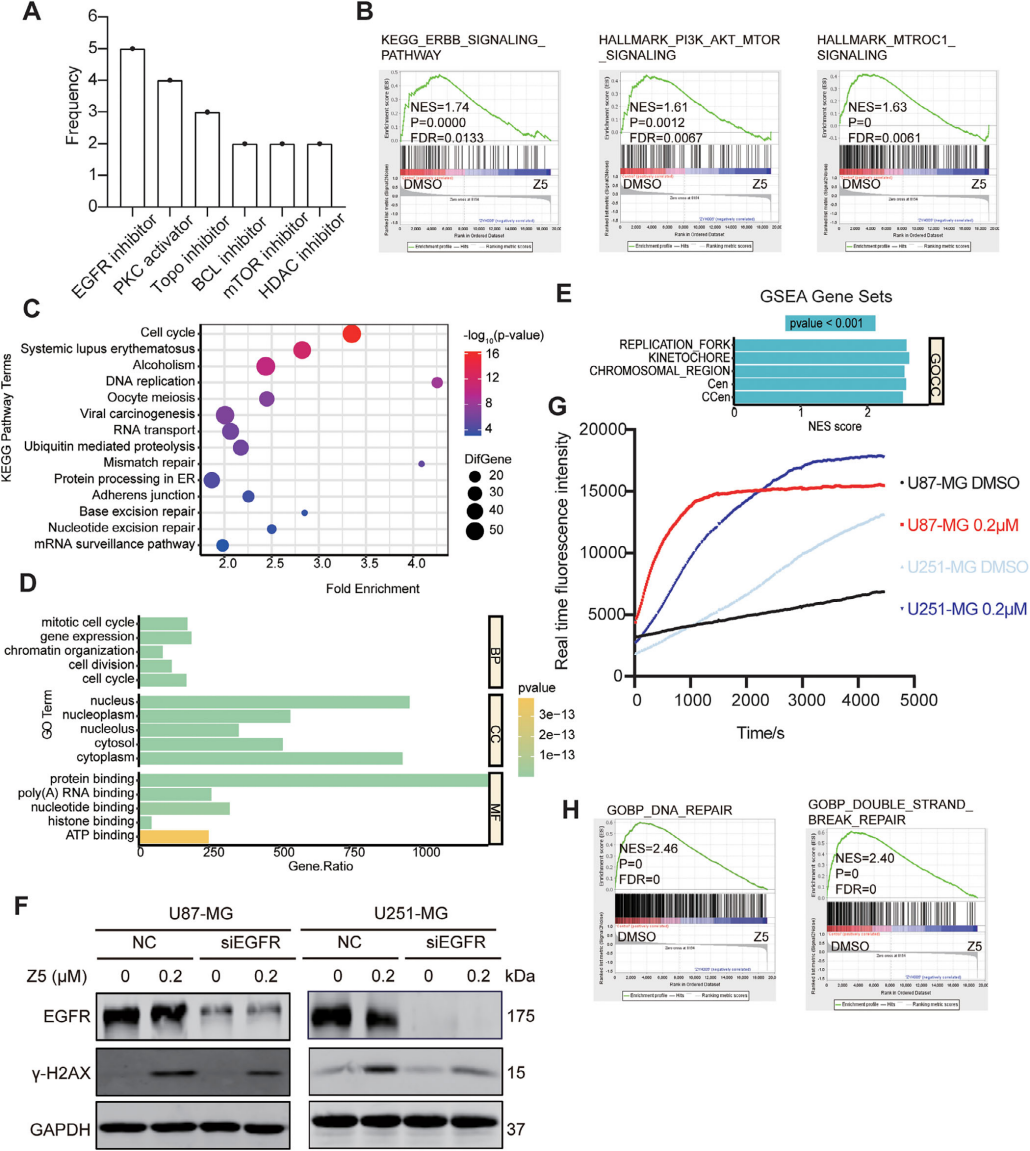
Z5 induces DNA damage through DNA intercalation and EGFR inhibition. (A) From CMap data, the top 10 compounds positively correlated with microarray profiles were selected per cell line. Compound descriptions were statistically analyzed and ranked by term frequency. (B) GSEA of the ERBB signaling pathway, PI3K/AKT/mTOR signaling and mTORC1 signaling. (C) KEGG pathway enrichment analysis of microarray data from GBM cells. (D)Top 5 enriched GO terms among downregulated genes in U87‐MG cells. (E) GSEA analysis of all genes identified by the U87‐MG microarray. Cen: chromosome centromeric region; CCen: condensed chromosome centromeric region. (F) EGFR and γ‐H2AX expression in NC and si‐EGFR‐1261 GBM cells was analyzed by immunoblotting after 48 h Z5 treatment. (G) Increased APE1‐accessible DNA damage in Z5‐treated cells. (H) GSEA of the DNA repair and double‐strand break repair.

We next interrogated the microarray data using the Connectivity Map (CMap) database. Querying CMap with a signature comprising the top 66 upregulated and top 150 downregulated genes from our microarray data yielded positive connectivity scores for multiple compounds. We categorized the data by cell type and selected only samples treated with inhibitor perturbations to identify the top 10 most effective inhibitors in each cell type. The results showed that EGFR inhibitors appeared most frequently, indicating that the transcriptional impact of Z5 treatment resembles that of multiple known EGFR inhibitors (Figure 4A). This conclusion was further supported by Gene Set Enrichment Analysis (GSEA): Z5 significantly suppressed the activation of ERBB signaling, PI3K/AKT/mTOR pathway and mTORC1 activity in U87‐MG cells (Figure 4B)—all of which are established pharmacological effects of EGFR inhibition. Together, these data demonstrate that the effect of Z5 on GBM cells is strongly associated with EGFR suppression, further confirming that Z5 acts as a targeted EGFR inhibitor.

KEGG enrichment analysis showed that Z5 could significantly affect DNA replication pathways, and Cellular Component (CC) enrichment analysis from GO and GSEA further revealed significant enrichment of dysregulated genes involved in nuclear compartments, replication forks, and chromosomal regions following Z5 treatment (Figure 4C–E), consistent with its DNA intercalating properties [19]. In line with these findings, Z5 treatment induced significant DNA damage in GBM cells, as evidenced by increased APE1‐mediated cleavage events—detected using a dual‐amplification fluorescent biosensor [26]—and elevated γH2AX foci indicating double‐strand breaks (Figure 4F,G). These findings suggest that the nucleus serves as a primary site for Z5's mechanism of action.

It is well‐established that nuclear EGFR plays a critical role in the DDR and that EGFR inhibition impairs DDR, resulting in the accumulation of DNA damage. In line with this, GSEA showed significant downregulation of DNA repair pathways after Z5 treatment (Figure 4H; Figure S4D), confirming that Z5 acts as an EGFR inhibitor to suppress the DDR. To further examine whether EGFR contributes to Z5‐induced DNA damage, we knocked down EGFR using siEGFR‐1261. First, we found that EGFR knockdown alone—despite its potential to suppress DDR—did not induce DNA damage in the absence of Z5 treatment. Second, EGFR knockdown reduced Z5‐induced DNA damage compared with wild‐type cells (Figure 4F), indicating that EGFR plays a functional role in Z5‐mediated DNA damage. Specifically, EGFR inhibition by Z5 acts synergistically to amplify DNA damage initiated through its primary mechanism of DNA intercalation. Together, these results demonstrate that Z5 induces DNA damage via a dual mechanism involving both DNA intercalation and EGFR inhibition.

### 2.5 Z5 induces G2/M Arrest and WEE1‐Mediated Mitosis Catastrophe Through EGFR Inhibition

**FIGURE 5:**
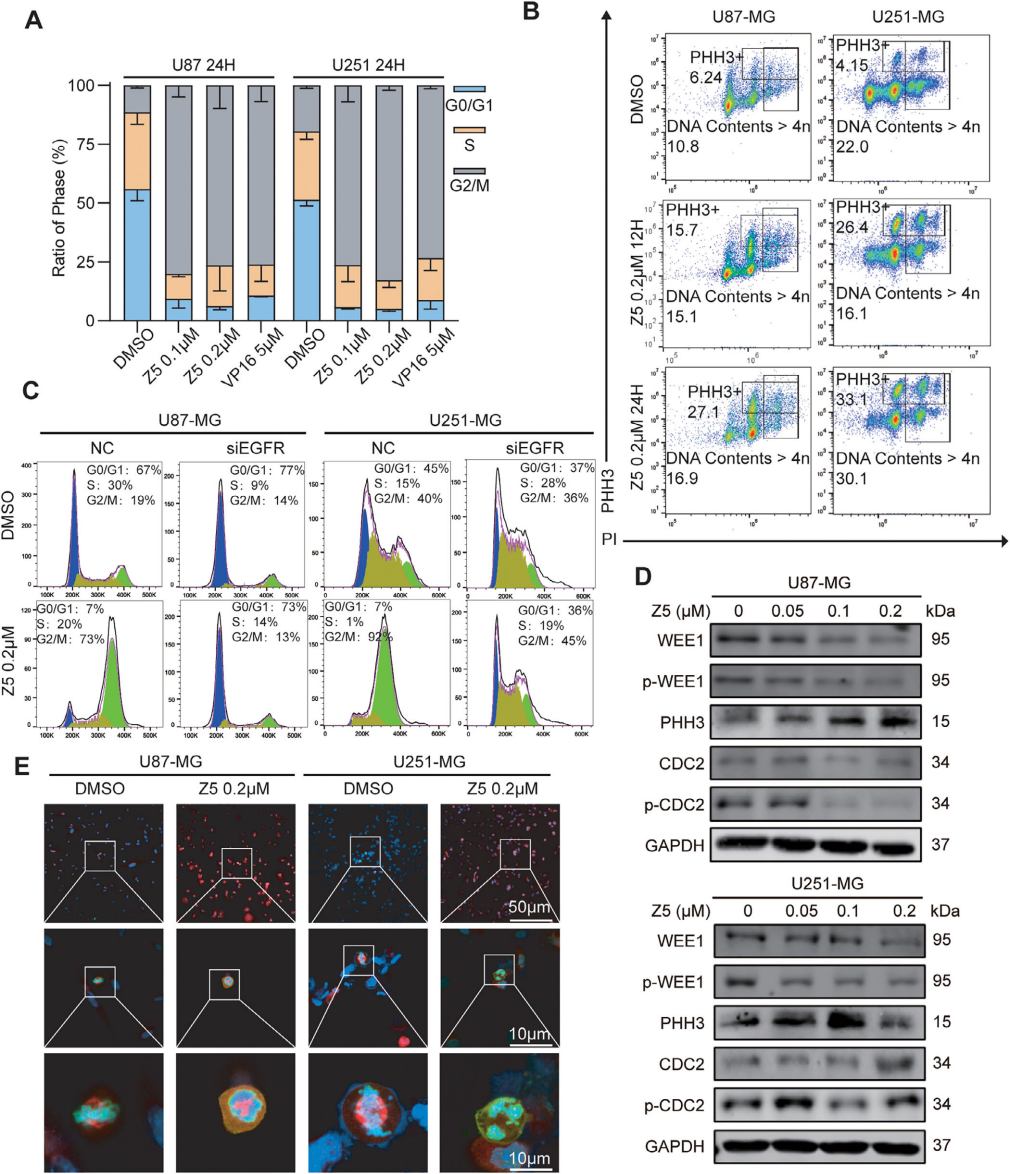
Z5 induces G2/M arrest and mitotic catastrophe in GBM cells. (A) Cell cycle distribution in GBM cell lines was analyzed by flow cytometry after 24 h of Z5 treatment. *n* = 3. (B) Representative flow cytometric analysis of PHH3+ and DNA contents > 4n cells. (C) Cell cycle profiles of NC and si‐EGFR GBM cells were assessed by flow cytometry after 24 h Z5 treatment. (D) Expression of WEE1, p‐WEE1, PHH3, CDC2, and p‐CDC2 was analyzed by immunoblotting after 48 h treatment with increasing concentrations of Z5. (E) IF staining was performed with anti‐tubulin (red) and anti‐PHH3 (green) antibodies in U87‐MG and U251‐MG cells treated with DMSO or Z5.

Following DNA damage, cells typically initiate cell cycle arrest to allow time for repair. Consistent with this mechanism, KEGG and GO analyses indicated that Z5 predominantly affects pathways related to cell cycle regulation and mitotic progression (Figure 4C,D). Subsequent GSEA using the GO‐BP, HALLMARK, and KEGG databases further underscored significant enrichment of terms associated with G2/M checkpoint signaling, G2/M phase transition, cell cycle control, and mitotic spindle assembly (Figure S4E). These findings suggest that Z5 disrupts the tightly regulated G2/M transition process. Consistent with these findings, we observed a robust arrest in the G2/M phase by flow cytometry (Figure 5A). Notably, Z5 treatment markedly elevated the populations of PHH3‐positive and polyploid cells (Figure 5B), a hallmark of mitotic catastrophe that is consistent with checkpoint failure. In addition, we found that EGFR knockdown completely abolished Z5‐induced G2/M arrest, demonstrating that Z5‐mediated cell cycle blockade is strictly dependent on EGFR inhibition (Figure 5C). To elucidate how Z5 disrupts the G2/M checkpoint through EGFR, we analyzed DEGs from microarray data. Among key regulators of the G2/M checkpoint, WEE1 showed the most pronounced downregulation (67%), while CDC2 (also known as CDK1)—which is phosphorylated by WEE1 to regulate mitotic entry—was also substantially reduced (55%) (Table S1), suggesting transcriptional disruption of the WEE1‐mediated checkpoint by Z5. Corroborating these findings, immunoblot analysis showed decreased phosphorylation of WEE1 and CDC2, along with reduced total protein levels of both WEE1 and CDC2 (Figure 5D). The decline in phosphorylation is likely a direct consequence of this downregulation: diminished WEE1 abundance limits the pool available for activating phosphorylation by upstream kinases (e.g., CHK1), while reduced CDC2 levels—combined with loss of its upstream kinase WEE1—collectively impair inhibitory phosphorylation at Tyr15. Concurrently, PHH3 expression was upregulated, indicating aberrant mitotic entry. Furthermore, immunofluorescence (IF) assays unveiled hallmark morphological features of mitotic catastrophe, such as chromosomal missegregation, multipolar spindle formation, and micronucleation (Figure 5E). Collectively, these results demonstrated that Z5 induces G2/M arrest and mitotic catastrophe through EGFR‐mediated inhibition of the WEE1 checkpoint.

### 2.6 Z5 targets Nuclear EGFR to Disrupt the EGFR–WEE1 Axis

**FIGURE 6:**
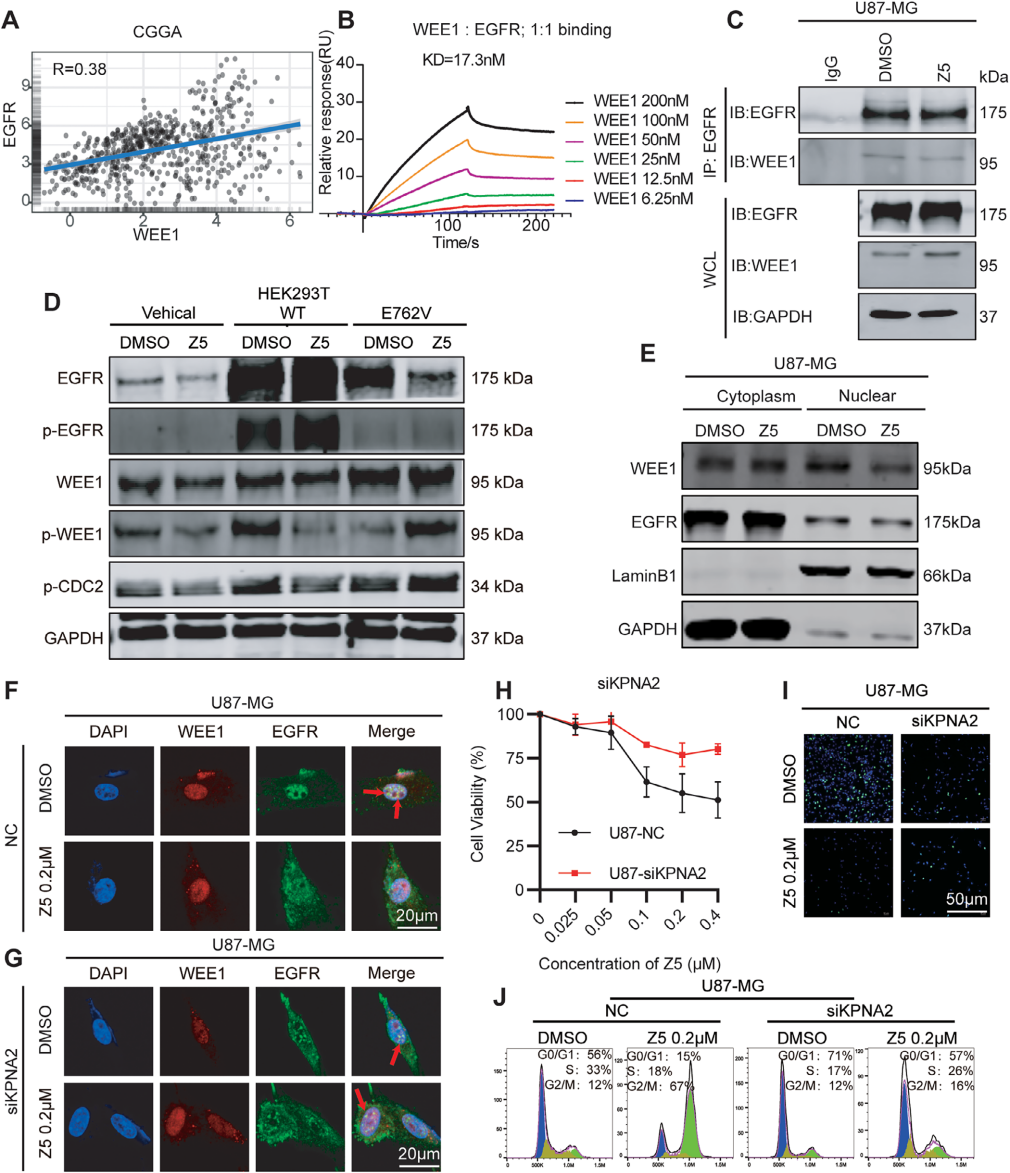
Z5 disrupts the nuclear EGFR–WEE1 axis. (A) Correlation between EGFR and WEE1 expression in GBM patients. (B) SPR analysis of the interaction between the kinase domains of EGFR and WEE1. (C) Co‐IP was performed to assess EGFR–WEE1 interaction in U87‐MG cells after 15 min Z5 treatment. (D) Western blot analysis of WEE1, p‐WEE1, p‐CDC2, EGFR, and p‐EGFR in HEK293T cells transfected with empty vector, WT‐EGFR, or E762V‐EGFR. (E) Subcellular localization of EGFR and WEE1 in the cytoplasm and nucleus was analyzed in U87‐MG cells after 15 min Z5 treatment. (F‐G) IF staining of EGFR and WEE1 in NC and siKPNA2‐1400 U87‐MG cells. (H) Growth curves of NC and siKPNA2‐1400 U87 cells after 48 h Z5 treatment. *n* = 3. (I) Antiproliferative effect of Z5 was assessed by EdU staining in NC and siKPNA2‐1400 U87‐MG cells. (J) Cell cycle distribution was analyzed by flow cytometry in NC and siKPNA2‐1400 U87‐MG cells after 24 h Z5 treatment.

To investigate whether the anti‐GBM efficacy of Z5 involves functional crosstalk between EGFR and WEE1, we first analyzed their correlation in glioma transcriptomes. Data from the Chinese Glioma Genome Atlas (CGGA) revealed a significant positive correlation between EGFR and WEE1 mRNA expression (Pearson's *R* = 0.38; Figure 6A). To determine whether this co‐expression reflects direct molecular interaction, we performed SPR analysis, which demonstrated high‐affinity binding between the kinase domains of EGFR and WEE1 with an equilibrium dissociation constant (KD) of 17.3 nM (Figure 6B). This interaction was further confirmed by co‐immunoprecipitation (Co‐IP) in GBM cells. Importantly, Z5 treatment significantly disrupted the EGFR–WEE1 complex, as shown by reduced Co‐IP of both proteins (Figure 6C; Figure S5A).

Next, we performed genetic and pharmacological perturbations in U87‐MG cells to dissect the functional relationship between EGFR and WEE1. Knockdown of WEE1 did not affect EGFR expression or phosphorylation. In contrast, EGFR knockdown significantly reduced both total WEE1 and phospho‐WEE1 levels, establishing EGFR as an upstream regulator of WEE1 (Figure S5B). Consistent with these findings, the WEE1 inhibitor MK1775 had minimal impact on EGFR signaling, whereas the EGFR inhibitor AZD9291 strongly suppressed both p‐WEE1 and p‐CDC2. These results confirm a unidirectional regulatory axis from EGFR to WEE1 (Figure S5C). Building on this established EGFR to WEE1 signaling relationship, we further investigated whether Z5 exerts its effects through direct interaction with EGFR. In 293T cells expressing the E762V mutant EGFR, Z5‐induced downregulation of p‐WEE1 and p‐CDC2 was markedly attenuated compared with wild‐type controls (Figure 6D). This indicates that Z5 targets EGFR via the E762 residue to inhibit its activity and disrupt the EGFR–WEE1 regulatory axis.

Since EGFR undergoes nucleocytoplasmic shuttling and WEE1 is predominantly nuclear, we hypothesized that their interaction is nuclear‐specific. To test this, we assessed their co‐localization. In control (DMSO‐treated) cells, both IF and cellular fractionation confirmed substantial nuclear co‐localization of EGFR and WEE1. Z5 treatment, however, disrupted this co‐localization, resulting in the dissociation of both proteins and their accumulation in the cytoplasm (Figure 6E,F; Figure S5D).

We next investigated whether the nuclear import of EGFR is essential for the anti‐GBM effects of Z5. Given that KPNA2 and KPNB1 facilitate EGFR nuclear translocation [27], we knocked KPNA2 down using a specific siRNA (siKPNA2‐1400) and confirmed a subsequent reduction in nuclear EGFR levels (Figure 6G; Figure S5E). This intervention significantly attenuated the ability of Z5 to inhibit cell proliferation (as assessed by CCK‐8 and EdU assays; Figure 6H,I, Figure S5F) and to induce G2/M phase arrest (Figure 6J; Figure S5G). The critical role of nuclear import was further emphasized when pharmacological inhibition of KPNB1 with importazole synergized with Z5 to further diminish its efficacy (Figure S5F). Importantly, the partial antiproliferative effect that remained after KPNA2 knockdown suggests that Z5's mechanism of action also involves pathways dependent on cytoplasmic and membrane‐localized EGFR. This finding is also in line with our previous detection of Z5's inhibition of the EGFR‐downstream mTOR and ERK pathways.

Given the limited exploration of the EGFR–WEE1 interaction in GBM, especially within clinical samples, we sought to address this gap by conducting an in‐depth analysis of transcriptomic data from GBM patient cohorts. This analysis aimed to evaluate the clinical relevance of this axis and to assess the translational potential of Z5. In the CGGA database, WEE1 expression was significantly elevated in GBM compared with other glioma subtypes (Figure S6A), with classical and mesenchymal subtypes showing higher levels than the proneural subtype (Figure S6B). IDH‐wildtype GBM exhibited stronger WEE1 expression relative to IDH‐mutant tumors (Figure S6C), consistent with a more aggressive phenotype. Survival analyses using TCGA and CGGA datasets revealed that high WEE1 expression was associated with poorer overall survival in GBM patients (Figure S6D–F). At the protein level, immunohistochemical staining of a tissue microarray containing 64 primary GBM samples (grades 0–IV) showed a positive correlation between WEE1 intensity and tumor grade (Figure S6G,H). IF further confirmed colocalization of EGFR and WEE1 in GBM tissues (Figure S6I) supports their functional interaction in vivo.

To summarize, we established that EGFR acts as an upstream regulator of WEE1 and revealed their specific nuclear interaction as a critical vulnerability. Z5 binds to EGFR, disrupts the nuclear EGFR–WEE1 complex, and consequently inhibits downstream signaling, leading to cell cycle arrest and suppressed tumor growth. The clinical relevance of this axis, supported by its association with aggressive tumor subtypes and poorer patient survival, highlights the central role of the EGFR–WEE1 axis in GBM pathogenesis and the translational potential of Z5.

### 2.7 Z5 suppresses GSCs Tumorigenesis and Inhibits EGFR‐Related Signaling in Vivo

**FIGURE 7:**
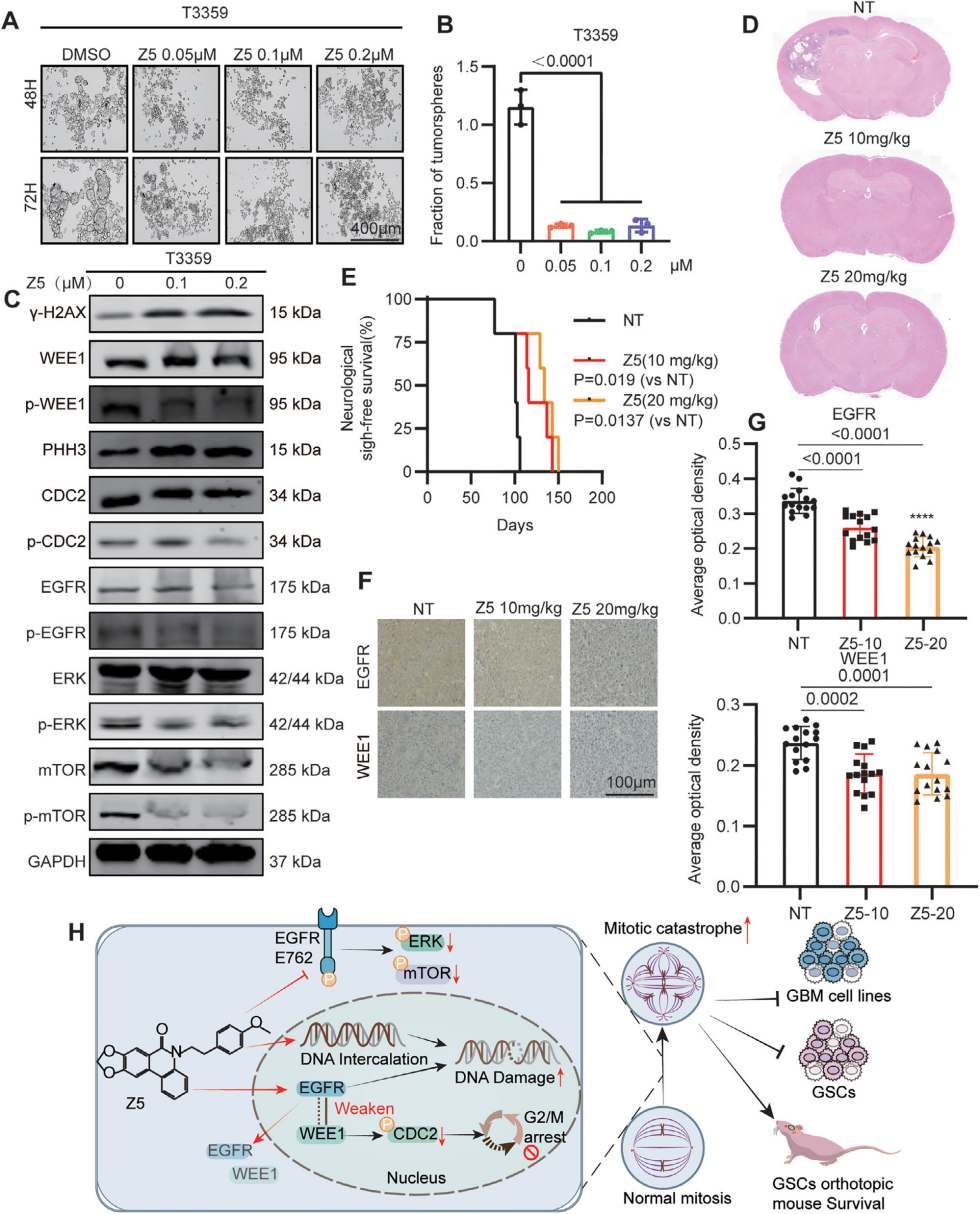
Z5 suppresses GSCs tumorigenesis and inhibits EGFR‐related signaling in vivo. (A) Representative tumor sphere formation assay in T3359 cells treated with different concentrations of Z5 for 48 h. Scale bar = 400 µm. (B) Quantitative analysis of tumor sphere fragmentation from (A). The data are presented as the mean ± SD, *n* = 3, compared with the “0” group. (C) Expression of γ‐H2AX, WEE1, p‐WEE1, PHH3, CDC2, p‐CDC2, EGFR, p‐EGFR, ERK, p‐ERK, mTOR, and p‐mTOR in T3359 cells with DMSO or Z5 treatment. (D) H&E staining of intracranial xenografts to assess tumor formation. (E) Survival analysis of mice orthotopically implanted with GSCs. The data are presented as the mean ± SD, *n* = 4, compared with the NT group. (F) Immunohistochemical analysis of EGFR and WEE1 expression in orthotopic tumor tissues. (G) Quantification of EGFR and WEE1 levels by average optical density from five fields per mouse. The data are presented as the mean ± SD, *n* = 15, compared with the NT group. (H) Schematic diagram illustrating the mechanism of Z5.

GSCs represent a distinct subpopulation within GBM, defined by their stem‐like properties and enhanced DNA repair capacity [28, 29]. Given their critical role in treatment resistance and tumor recurrence, we chose this most challenging and representative model to further assess Z5's anti‐GBM effects. Accordingly, we performed a tumor sphere formation assay, which revealed that Z5 markedly induced fragmentation of tumor spheres derived from the T3359 and D456 GSCs (Figure 7A,B, Figure S7A,B). Immunoblotting further confirmed that Z5 significantly suppressed the EGFR–WEE1 axis in these cells (Figure 7C, Figure S7C). Notably, Z5 exhibited stronger inhibitory effects in T3359 than in D456, and therefore, T3359 was selected for subsequent in vivo experiments to evaluate antitumor efficacy.

Then, we established an orthotopic xenograft model. Upon terminal symptom onset in the first untreated (NT) mouse, one mouse per treatment group was euthanized for histological analysis. HE staining showed that Z5 markedly suppressed intracranial GSCs tumor growth (Figure 7D). Z5 also prolonged survival in a dose‐dependent manner. Mice in the NT group survived for a median of 102.75 days, whereas those treated with 10 and 20 mg/kg Z5 exhibited median survivals of 127.5 and 139 days, corresponding to 24.1% and 35.3% increases, respectively (Figure 7E). Immunohistochemistry (IHC) staining further demonstrated reduced EGFR and WEE1 expression in Z5‐treated tumors (Figure 7F,G). Notably, after 8 weeks of treatment, H&E staining of heart, liver, spleen, lung, and kidney tissues revealed no toxicity (Figure S8A–E). These findings establish Z5 as a potent inhibitor of GSCs growth in both in vitro and in vivo settings, accompanied by effective suppression of the EGFR–WEE1 axis and a favorable safety profile.

In conclusion, Z5 exerts potent anti‐GBM efficacy via a dual mechanism: DNA intercalation that induces damage, and targeted inhibition of EGFR at E762. The DNA damage response is amplified by EGFR inhibition, which concurrently disrupts the EGFR–WEE1 interaction. This disruption compromises WEE1‐mediated regulation of its downstream effector CDC2, leading to reduced p‐CDC2 levels, G2/M checkpoint failure, mitotic aberrations, and ultimately, mitotic catastrophe. Furthermore, Z5 inhibits the mTOR and ERK pathways, confirming the suppression of plasma membrane‐localized EGFR signaling as a key contributor to its efficacy. Collectively, based on these mechanisms, Z5 demonstrates potent antitumor activity across several GBM models, including established cell lines, patient‐derived GSCs, and orthotopic xenografts, underscoring its considerable therapeutic potential (Figure 7H).

## 3 Discussion

Since TMZ's approval in 2005, despite extensive research over the past 2 decades, no transformative therapies have emerged. Currently, the top‐priority task in this field is to spare no effort in the search for new molecules and methods that are effective against GBM. Addressing this critical gap, our study successfully identified Z5 as a novel, dual‐functional EGFR inhibitor that not only demonstrates potent antitumor efficacy across a spectrum of preclinical GBM models but also exhibits a favorable safety profile, distinguishing it from many existing therapeutic candidates. More importantly, we have systematically elucidated its unique mechanism of action, revealing that Z5 achieves its robust effects by inducing DNA damage through intercalation and precisely targeting the EGFR signaling axis at the E762 residue, thereby disrupting the critical EGFR–WEE1 regulatory circuit and downstream survival pathways. This comprehensive investigation positions Z5 as a highly promising candidate worthy of further clinical development, offering a new strategic approach to combat this devastating disease.

We have previously established Z5 as a DNA intercalator [19]. Z5 exhibits moderate intercalation strength compared with classical intercalators such as YOYO‐1 and ethidium bromide. This property may correlate with reduced carcinogenic and mutagenic potential, as supported by in vitro and in vivo toxicity studies in normal cells and mouse models. Single‐molecule magnetic tweezer experiments also revealed that Z5 induces only moderate changes in DNA extension and twist under low force conditions, which may account for its potent antitumor activity coupled with low systemic toxicity [19]. These findings were further corroborated in the present study through long‐term toxicity assessment over 150 days in an orthotopic GBM model using patient‐derived GSCs. Given its high safety profile, further investigation into its full range of biological effects and underlying mechanisms is warranted.

Despite the introduction of novel therapeutic modalities such as tumor‐treating fields, molecularly targeted agents, and immunotherapies, the clinical management of GBM remains profoundly challenging. These approaches are consistently hampered by tumor heterogeneity, an immunosuppressive microenvironment, and—critically—the limited penetration of the BBB, which collectively contribute to transient responses and recurrence rates exceeding 90% [30, 31]. In particular, conventional EGFR inhibitors (e.g., gefitinib, osimertinib) exhibit restricted efficacy in GBM, owing not only to compensatory signaling and tumor adaptability but also to their inadequate BBB permeability [32, 33, 34]. Z5, as a novel phenanthridine alkaloid derivative, has shown remarkable BBB penetration and potent antitumor efficacy, providing further evidence for the ability of this structural class to cross the BBB and exert therapeutic effects in brain diseases. Consistently, natural phenanthridine alkaloids such as narciclasine and lycorine have been confirmed by previous studies to exhibit significant anti‐GBM activity, while lycorine, galanthamine, and haemanthamine have also demonstrated neuroprotective effects [35, 36, 37]. Of particular note, galanthamine, a representative phenanthridine alkaloid and a natural product from the *Amaryllidaceae* family, has been approved by the FDA for the treatment of Alzheimer's disease. Its successful clinical application serves to underscore the potential of this chemical family to cross the BBB and treat neurological disorders, thereby offering a compelling rationale and considerable promise for the translational research of Z5.

DNA intercalators reversibly insert between adjacent base pairs of double‐stranded DNA, thereby altering DNA conformation, compromising structural integrity, and interfering with DNA‐processing proteins [38, 39]. Unlike alkylating agents, which exert anticancer effects through irreversible covalent modification of DNA, intercalators bind via noncovalent interactions—primarily van der Waals forces, hydrogen bonding, hydrophobic effects, and/or charge‐transfer interactions—resulting in reversible DNA binding [40]. Consequently, their binding does not necessarily induce immediate cytotoxicity. To elicit cell‐killing effects, intercalators must first form a sufficiently stable complex with a long half‐life, capable of obstructing DNA‐metabolizing enzymes and impairing essential processes such as replication, transcription, and translation. Classic examples include anthracycline drugs like doxorubicin and daunorubicin, which intercalate into DNA but primarily cause cytotoxicity by inhibiting topoisomerase II, preventing DNA religation, and leading to irreversible strand breaks. In this study, we employed multiple experimental approaches to demonstrate that, beyond its DNA‐intercalating activity, Z5 also functions as a potent EGFR inhibitor. By specifically targeting the E762 residue of EGFR, Z5 effectively suppresses canonical plasma membrane‐derived signaling pathways—such as mTOR and ERK—and concurrently impairs EGFR‐mediated DNA damage repair, resulting in the accumulation of unresolved DNA lesions. Importantly, this strategy represents a significant advance over monotherapeutic approaches that focus exclusively on nuclear EGFR or individual downstream effectors [41, 42].

Interestingly, the extent of EGFR pathway suppression by Z5 varied between GBM models. In U87‑MG, Z5 induced a more pronounced downregulation of EGFR, p‑ERK, p‑mTOR, and p‑CDC2 than in U251‑MG, consistent with intrinsic molecular heterogeneity between these lines. U87‑MG harbors wild‑type TP53, whereas U251‑MG carries the hotspot mutation R273H in TP53 [43]. In the wild‑type context, Z5‑induced DNA damage and EGFR inhibition may activate p53, triggering p21‑mediated cell‑cycle arrest and attenuating pro‑survival signaling. By contrast, mutant TP53 (R273H) in U251‑MG has been reported to repress miR‑27a, resulting in elevated EGFR expression and sustained ERK activation [44]. Although this axis remains unvalidated specifically in U251‑MG, a comparable mechanism could account for the comparatively stable EGFR levels and weaker pathway suppression observed after Z5 treatment. Critically, our data reveal a strong correlation between the efficacy of Z5 and its ability to downregulate total EGFR protein levels. In models where Z5 failed to substantially reduce EGFR protein abundance (e.g., U251‑MG and D456), therapeutic potency was markedly attenuated. This observation suggests that while Z5 effectively inhibits EGFR kinase activity, certain GBM cells may sustain survival not only through kinase‐dependent signaling but also via kinase‐independent functions mediated by the physical presence of the EGFR protein itself [5]. Consequently, mere inhibition of kinase activity without concomitant protein degradation may be insufficient in these contexts. These findings carry significant clinical implications: EGFR amplification or wild‐type status alone may not serve as reliable positive predictors for response to EGFR tyrosine kinase inhibitors. Instead, clinical stratification should adopt a more comprehensive approach, integrating TP53 status and other biomarkers that influence EGFR protein stability and noncanonical functions to better identify patients likely to benefit from such targeted therapies.

Beyond the distinct molecular profiles of established cell lines, the complexity of GBM is further exemplified by patient‐derived GSCs. In our subtype analysis of T3359 and D456, while both lines exhibited the highest scoring for the CL subtype, they simultaneously displayed significant transcriptional signatures characteristic of the PN and MES subtypes. This co‐expression of multiple subtype features underscores the fundamental nature of GSCs, which are defined by their capacity for self‐renewal and multipotent differentiation into various lineages within the tumor hierarchy [45]. Recent single‐cell RNA sequencing studies have corroborated this observation, revealing that individual GBM tumors are not monolithic entities but rather contain multiple co‐existing cellular states that map to these canonical subtypes [29]. Crucially, these findings suggest that GBM subtypes primarily reflect the relative abundance of specific cellular states rather than their exclusive presence. Such intrinsic plasticity enables GSCs to dynamically adapt to therapeutic pressures, playing a pivotal role in tumor recurrence and drug resistance. Consequently, our results highlight that subtype‐specific therapeutic strategies for GBM must be approached with caution.

WEE1 plays a critical role in maintaining genomic stability by phosphorylating CDK1/2 to enforce the G2/M checkpoint, thereby allowing DNA repair before mitotic entry [46]. Its overexpression is frequently associated with poor prognosis and therapy resistance in multiple cancers, while pharmacological or genetic inhibition of WEE1 can induce mitotic catastrophe in genomically unstable tumor cells [47]. In GBM, where EGFR amplification and replication stress are highly prevalent, WEE1 serves as a key survival factor that helps cancer cells cope with DNA damage and replication pressure [48]. In this study, we systematically delineated, for the first time, the functional interplay between EGFR and WEE1 in GBM. We demonstrated that Z5 disrupts this EGFR–WEE1 axis, thereby compromising the protective checkpoint mechanism and driving GBM cells into mitotic catastrophe. These findings not only reveal a novel mode of action for Z5 but also provide compelling evidence supporting the EGFR–WEE1 axis as a therapeutically targetable vulnerability in GBM. Z5 represents both a mechanistic probe for understanding EGFR–WEE1 biology and a promising therapeutic candidate worthy of further development.

While our study elucidates a novel mechanism of action for Z5, several questions remain unresolved. First, it is still unclear whether the disruptive effects of Z5 on the EGFR–WEE1 interaction are mediated solely through its EGFR‐targeting activity, independently of DNA intercalation, or via a combined mechanism. Second, the structural basis of the EGFR–WEE1 interaction—particularly the precise binding interface—requires further elucidation [9, 48, 49]. Third, it remains to be determined whether Z5 inhibits EGFR tyrosine kinase activity and how its efficacy compares with that of existing EGFR inhibitors. Fourth, although our preclinical data indicate that Z5 is well tolerated in vivo, with no evident organ toxicity or neurological deficits observed in mice under long‑term administration, the potential impact of Z5 on normal cells of the brain microenvironment—including astrocytes, neurons, and brain endothelial cells—was not directly assessed. Given the critical role of these cells in maintaining neural homeostasis and the blood–brain barrier, their vulnerability to Z5 warrants systematic investigation in future studies. Fifth, our current investigation primarily utilized GSC models enriched for CL signatures. The absence of dedicated PN and MES subtype models limits our ability to fully assess Z5's efficacy across the entire spectrum of GBM heterogeneity. Future studies incorporating a broader panel of subtype‐specific GSCs are essential to validate the pan‐subtype therapeutic potential of Z5. These aspects are critical for optimizing the therapeutic application of Z5 and warrant further investigation.

In conclusion, Z5 demonstrates potent anti‐GBM efficacy across classical subtypes by concurrently targeting DNA and EGFR, thereby inducing mitotic catastrophe while maintaining a favorable safety profile. This study establishes Z5 not only as a mechanistic probe for EGFR signaling but also as a brain‐penetrant clinical candidate, and reveals disruption of the nuclear EGFR–WEE1 axis as a novel therapeutic vulnerability in GBM. These findings provide a strong foundation for advancing Z5 toward clinical translation and offer a promising new strategy for classical GBM treatment.

## 4 Materials and Methods

### 4.1 Compounds and Cell Lines

Z5 was synthesized in our laboratory, and its synthesis process has been described previously [19]. The U87‐MG (RRID: CVCL_0022), U251‐MG (RRID: CVCL_0021), and HEK293T (RRID: CVCL_0063) cell lines were purchased from Procell Life Science & Technology. GSCs (T3359 and D456) were provided by Dr. Shideng Bao and Dr. Jeremy Rich [50]. U87‐MG, U251‐MG and HEK293T cells were cultured in DMEM (Pricella, Wuhan, China) supplemented with 10% fetal bovine serum (FBS) and 1% penicillin/streptomycin. GSCs were cultured in a serum‐free system containing Neurobasal A basal medium supplemented with B‐27 supplement minus vitamin A, L‐glutamine, sodium pyruvate, 20 ng/mL recombinant epidermal growth factor, and 20 ng/mL basic fibroblast growth factor. The cell culture, cryopreservation, and thawing procedures were all meticulously conducted in strict accordance with standard operating procedures (SOPs). Short tandem repeat (STR) profiling and contamination assessment of the cell lines were performed every six months. The results verified the authenticity of the cell lines and confirmed their contamination‐free status.

### 4.2 DNA Microarrays and Gene Set Enrichment Analysis

Total RNA was extracted from U87‐MG cells using TRIzol reagent (Cat# 15596‐018, Life Technologies, Carlsbad, CA, USA). RNA purification was performed using the NucleoSpin RNA Clean‐up XS kit (Cat#740903, MN, Germany) and the RNase‐Free DNase Set (Cat#79254, QIAGEN, GmbH, Germany). According to the Affymetrix technical manual, the double‐stranded cDNA was hybridized with biotin‐labeled fragmented cRNA. Microarray experiments were performed using Affymetrix chips by Shanghai Biotechnology Corporation. The raw data were normalized via the expression console algorithm in Command Console Software 4.0 (Affymetrix, Santa Clara, CA, USA). Differential expression analysis was conducted via the affy package in R. Genes with an absolute log2‐fold change >1 and a *p*‐value <0.05 were considered differentially expressed. The microarray data for U87‐MG cells have been submitted to the NGDC/OMIX under accession number OMIX008765. Significantly differentially expressed genes (|Log2FC| ≥ 1, FDR ≤ 0.05) are listed in Table S1. All genes identified through DNA microarray analysis were imported into the GSEA software for further analysis and visualization.

### 4.3 Molecular Docking of Z5 With the EGFR Kinase Domain

For our molecular docking experiments, we utilized the crystal structure of EGFR (PDB ID 7KXZ) [51]. This structure was preprocessed via the PDB2PQR program [52] to determine the protonation state of titratable residues at pH = 7 and subsequently converted to pdbqt format via the prepare_receptor4.py script from AutoDockTools [53]. Next, we prepared Z5 by converting the compounds to pdbqt format (pH 7.0) via Open Babel. Molecular docking scores were used as indicators of the predicted binding affinity.

### 4.4 Limited Proteolysis–Mass Spectrometry

LiP–MS was performed as previously published [54]. A detailed description is provided in the Supporting Information Material.

### 4.5 Tissue Microarray and Immunohistochemistry

Tissue microarrays (TMA) were obtained from Wuhan Powerful Biology Company. The use of human tissues in this study was approved by the Ethics Committee of Tongji Hospital of Tongji Medical College of Huazhong University of Science and Technology (Serial number: 2021‐lEC‐A244). TMA sections were deparaffinized and subjected to antigen retrieval prior to immunostaining. WEE1 antibody (Cell Signaling Technology, 1:200 dilution) was used to detect the protein in the GBM tissue samples.

IHC for in situ transplanted mouse brain tissues was carried out via the same protocol as described above. WEE1 antibody (Cell Signaling Technology, 1:200 dilution) and EGFR antibody (Proteintech, 1:100 dilution) were used to detect the protein.

### 4.6 Orthotopic Transplantation Mouse Model

Experimental BALB/c nude mice were purchased from Beijing Vital River Laboratory Animal Technology Co., Ltd. (Beijing, China) and kept at the Experimental Animal Center of Tongji Medical College, Huazhong University of Science and Technology, Wuhan, China. All the mice were anesthetized with phenobarbital sodium before surgery. One week after GSCs (2 × 10^4^ cells/mouse) were implanted in the right frontal lobe of 4‐week‐old mice, 15 mice were randomly divided into three groups: the untreated (NT), Z5 low‐dose (10 mg/kg), and Z5 high‐dose (20 mg/kg) groups. The mice received either solvent (5% DMSO, 20% castor oil, and 75% saline) or varying doses of the drug by oral gavage daily for 7 weeks. When the NT group showed the first manifestation of obvious neurological signs, one mouse from each of the remaining two groups was evaluated for drug efficacy. The remaining 12 mice were used for survival studies.

### 4.7 Statistical Analysis

Statistical analysis was performed via GraphPad Prism 9.0 software. The data are expressed as the mean ± SD. We used unpaired *t*‐tests for statistical analysis between two groups and one‐way ANOVA for multiple comparisons. All the experiments were repeated more than three times. We considered a *p*‐value less than 0.05 to indicate statistical significance.

## Author Contributions

Yonghui Zhang, Qingyi Tong, Suojun Zhang, and Xingjiang Yu: funding acquisition, resources. Jianzheng Huang and Qingyi Tong: data curation, writing – original draft, and writing – review editing. Zijun Zhang, Xiao Yang, and Ziming Zhao: Validation. Zengwei Luo, Junjun Liu, and Suitian Lai: visualization. Chao Song and Shouchang Feng: resources. All authors have read and approved the final manuscript.

## Funding Information

This work was financially supported by the National Natural Science Foundation of China [82073886, 82473132 and 82072805], the Program for Medical Youth Talent of Hubei Province (2024–2027), the Hubei Provincial Administration of Traditional Chinese Medicine Research Fund [ZY2023M063], and the National Key R&D Program of China [2021YFA0910500].

## Ethics Statement

GSC cells were obtained from glioma patients who underwent surgery at the Department of Neurosurgery of Tongji Hospital, Tongji Medical College of Huazhong University of Science and Technology. All participants provided written informed consent, and the study was approved by the Ethics Committee of Tongji Hospital of Tongji Medical College of Huazhong University of Science and Technology (Serial number: 2021‐lEC‐A244). The Animal Experiment Administration Committee of the Huazhong University of Science and Technology (HUST) approved all the animal experiments to ensure ethical and humane treatment of the animals (IACUC number: 3028).

## Conflicts of Interest

Author Yonghui Zhang is an Editorial board member of MedComm. Yonghui Zhang was not involved in the journal's review of or decisions related to this manuscript. ZYH005 is patent (ZL201911229652.1, Yonghui Zhang, Qingyi Tong, Zengwei Luo, Hucheng Zhu), but has no potential relevant financial or nonfinancial interests. The remaining authors declare no conflicts of interest.

## Supporting information




**Supporting File 1**: mco270717‐sup‐0001‐TablesS1‐S7.xlsx

**Supporting File 2**: mco270717‐sup‐0002‐SuppMat.docx

## Data Availability

This study did not generate new reagents or original code. All the data generated or analyzed during this study are included in this article and its supplementary information files. Data of Array (OMIX008765) can be downloaded from: https://share.cncb.ac.cn/IXO27oQq0w/OMIX008765/.
