# Supplementary material for: Dual Targeting of DNA and EGFR by ZYH005 Induces DNA Damage and Mitotic Catastrophe in Glioblastoma
Source: MedComm (2020). 2026 Apr 1;7(4):e70717. doi: 10.1002/mco2.70717 (PMC13045254; doi:10.1002/mco2.70717)
Supplement: Supplementary file 2 — Supporting File 2: mco270717‐sup‐0002‐SuppMat.docx [file MCO2-7-e70717-s001.docx]

**Supporting Information**

**Dual Targeting of DNA and EGFR by ZYH005 Induces DNA Damage and Mitotic Catastrophe in Glioblastoma**

**Running title:** **ZYH005 targets DNA/EGFR to inhibit glioblastoma**

Jianzheng Huang^1^, Zijun Zhang^1^, Yang Xiao^1^, Ziming Zhao^1^, Zengwei Luo^1^, Junjun Liu^1^, Suitian Lai^1^, Chao Song^2^, Shouchang Feng^3^, Suojun Zhang^3^, Xingjiang Yu^2^, Qingyi Tong^1*^, Yonghui Zhang^1*^

^1^Department of Hubei Key Laboratory of Natural Medicinal Chemistry and Resource Evaluation, School of Pharmacy, Tongji Medical College, Huazhong University of Science and Technology, Wuhan, 430030, China;

^2^Department of Histology and Embryology, School of Basic Medicine, Tongji Medical College, Huazhong University of Science and Technology, Wuhan, China;

^3^Department of Neurosurgery, Tongji Hospital, Tongji Medical College, Huazhong University of Science and Technology, Wuhan, China.

Corresponding Author: Yonghui Zhang, (zhangyh@mails.tjmu.edu.cn.); Qingyi Tong, ([qytong@hust.edu.cn](mailto:qytong@hust.edu.cn).);

1. **Materials and Methods**

**1.1 Histology analysis**

Brain tissues were fixed with 4% paraformaldehyde for 12 hours and embedded in paraffin. The paraffin-embedded brain specimens were sectioned and stained with H&E, according to the manufacturer’s protocol. Other sections of brain specimens were subjected to immunohistochemistry (IHC), and the samples were incubated with primary antibodies against WEE1 (Cell Signaling Technology) and EGFR (Abclonal) for 30 minutes at room temperature. Subsequently, the slides were incubated with secondary antibodies and hematoxylin.

**1.2 Determination of Blood-Brain Barrier (BBB) Penetration:**

To evaluate the BBB penetration of ZYH005 (Z5), twelve C57BL/6 mice were randomly divided into four groups: a vehicle control group and three treatment groups sacrificed at 0.25, 0.5, and 2 h after intraperitoneal administration of Z5. After injection, mice were anesthetized with isoflurane and blood samples (~0.5 mL) were collected by cardiac puncture into pre-chilled tubes containing EDTA-K₂ as an anticoagulant. Samples were kept on wet ice and centrifuged within 1 h of collection (1700 × g, 4 °C, 10 min) to obtain plasma.

Subsequently, mice were transcardially perfused with ice-cold saline (0.9% NaCl) until the liver appeared pale to remove intravascular blood. Brain tissues were then harvested and homogenized in a ratio of 1:3 (w/v; 1 g tissue to 3 mL water). The concentrations of Z5 in plasma and brain homogenate were quantified using liquid chromatography-tandem mass spectrometry (LC-MS/MS).

**1.3 Cell viability assay**

Cell viability was measured using the CCK-8 assay. Briefly, cells were seeded into 96-well plates at a density of 3,000 cells per well and allowed to adhere overnight before treatment with various concentrations of drugs or vehicle. At 24, 48, or 72 h post-treatment, 10 μL of CCK-8 reagent was added to each well and cells were incubated at 37°C for 4 h. The optical density (OD) was measured at 450 nm using a microplate reader. IC₅₀ values were determined by nonlinear regression analysis of dose-response curves using SPSS software

For drug combination studies, cells were co-treated with varying concentrations of Z05 and importazole for 48 h. Synergy scores were calculated using the SynergyFinder+ web application: [SynergyFinder+](https://synergyfinder.org/#!/).

**1.4 Clone formation assay**

Cells were seeded into 6-well plates at a density of 500 cells per well and allowed to adhere overnight. Cells were then treated with DMSO, Z5 or Temozolomide (TMZ) for 12 h, after which the drug-containing medium was replaced with fresh drug-free medium. The medium was changed every three days thereafter. The cells were collected after 14 days of culture.

**1.5 EdU staining**

EdU cell proliferation staining was performed using an EdU kit (BeyoClick™ EdU Cell Proliferation Kit with Alexa Fluor 488, Beyotime, China). The kit was used according to the manufacturer’s instructions.

**1.6 Immunoblotting assay**

Immunoblotting was performed as previously published^1^. The membranes were washed with TBST and incubated with the secondary antibodies anti-rabbit IgG (H+L) (CST, #5151, Boston, American) and anti-mouse IgG (H+L) (CST, #5257, Boston, American), followed by imaging using a Li-Cor Odyssey scanner (LI-COR, USA). Image analysis was executed using Image Studio software. Primary antibodies used in the study are listed in **Table S2**.

**1.7 DNA fragment detection assay**

Cells were seeded into 6-well plates and incubated with DMSO or Z5. After 24 hours, the cells were washed once with cold PBS. Genomic DNA was extracted using the Universal Genomic DNA Purification Mini Spin Kit, as per the manufacturer’s instructions. DNA fragment detection was performed as previously published ^2^.  Briefly, a mixture of DNA, TdT, and dATP was prepared and incubated at 37℃ for 30 minutes, followed by an additional incubation at 75℃ for 20 minutes. Finally, Endo IV and the probe were added to the system. The fluorescence intensity was measured immediately using the real-time PCR instrument (RRID: SCR_018018).

**1.8 Cellular Thermal Shift Assay (CETSA)**

After freeze-thawing the GBM cells twice using liquid nitrogen to get the whole protein, divide the protein solution into two equal parts. Incubate each part with DMSO or Z5 at room temperature for 1 hour. After incubation, aliquot the protein solution and incubate at different temperatures for three minutes. Following this, centrifuge the samples at 12000g, collect the supernatants, and perform immunoblotting analysis.

**1.9 Drug Affinity Responsive Target Stability Assay (DARTS)**

DARTS was performed as previously published^3^. Briefly, after lysing GBM cells with RIPA buffer, divide the protein solution into five equal parts, with each portion being 90 μL. Measure the protein concentration using the BCA kit. Meanwhile, incubate each portion of the protein solution with DMSO or different concentrations of Z5 at room temperature for 15 minutes. Based on the protein concentration, add protease solution at a ratio of 1:700 (enzyme: protein) and incubate at room temperature for 10 minutes. Subsequently, stop the reaction by adding a protease inhibitor cocktail and SDS-PAGE loading buffer followed by immunoblotting.

**1.10 Surface plasmon resonance (SPR)**

A Biacore 1K instrument was used to measure the binding kinetics of the human EGFR kinase domain (EGFR residues 704-1016) to Z5. Measurements were performed at 25℃. Proteins were dissolved in PBS, pH7.4. The proteins were immobilized on the CM5 sensor chip (Cytiva, 29149603, Shanghai, China) using the amine-coupling method according to standard protocols. Data were processed using standard double-referencing and fit to a 1:1 binding model using Biacore 1K Evaluation software. The association rate (K_on_, M^−1^ s^−1^), dissociation rate (K_off_, s^−1^), estimate of error (SE), Chi-Square (Chi^2^), and statistic and maximum response (R_max_) and response units (RU) were determined. The equilibrium dissociation constant (K_d_) was calculated from the relationship *K_D_* = K_off_/K_on_ (M). GraphPad Prism 9.0 was used for image processing.

**1.11 Transfection of siRNA and plasmids**

U87-MG and U251-MG cell lines were transfected with siRNA using siRNA-Mate (GenePharma, Suzhou, China). Transfections were carried out following the siRNA-Mate protocol. The siRNA sequences were shown in **Table S3**. The pcdna3.1 (Vehicle), EGFR WT and EGFR E762V plasmids were purchased from Gene Create (Wuhan, China). The plasmids were transfected into the HEK293T cells using Lipofectamine 3000 (Thermo Scientific, USA) according to the manufacturer’s instructions. The plasmid sequences were shown in **Table S4**.

**1.12 Limited Proteolysis–Mass Spectrometry (LiP-MS)**

Recombinant EGFR-kinase domain (EGFR residues 704-1016) were incubated with 100 μM of Z5 at 25°C for 15 minutes; Control Group: Recombinant EGFR-kinase domain were incubated with an equal volume of DMSO at 25°C for 15 minutes. After incubation, Proteinase K was added to the protein solutions (enzyme-to-protein ratio by weight is 1:100), and the mixture is digested at 25°C for 3 minutes, followed by immediate heat treatment at 98°C for 5 minutes to stop the enzymatic reaction. The samples, post-Proteinase K digestion, were cooled to room temperature, then an equal volume of 2% SDC (prepared in 20 mM Tris-HCl buffer) is added, adjusting pH to 7-8.5 with ammonium bicarbonate. The samples were heated at 98°C for 5 minutes, cooled to room temperature, and then 5 μL of 0.1 M TCEP and 5 μL of 0.4 M chloroacetamide were added, reacting in the dark at 45°C with shaking at 1500 rpm for 5 minutes. After cooling the samples to room temperature, trypsin (Promega, Madison, WI) was added at an enzyme-to-sample ratio (by weight) of 1:50 and incubated overnight at 37°C for proteolysis. An appropriate amount of formic acid was added to achieve a final concentration of 1.5%, mixed thoroughly, and centrifuged at 16000g for 5 minutes. The supernatant was collected, desalted using a C18 desalting column, and dried under vacuum. Each sample had been resuspended in 30 μL of Solvent A (A: 0.1% formic acid in water) to create a suspension. Then, 9 μL of the suspension was mixed with 1 μL of 10× iRT peptide mix. After mixing thoroughly, the samples were subjected to separation by nano-Liquid Chromatography (nano-LC). The separated peptides were analyzed by online electrospray tandem mass spectrometry (MS/MS). The differential peptides identified by LIP-MS can be found in **Table S5**. The full peptides identified by LIP-MS can be found in **Table S6**

**1.13 qPCR**

Total RNA was extracted from cells with Trizol and was transcribed to cDNA using HiScript QRT SuperMix reverse transcriptase (Vazyme, R223–01, Nanjing, China). qPCR was then performed on ABI QuantStudio 5 (Thermo Fisher Scientific, USA, RRID: SCR_018018) using SYBR Green qPCR Mix (Vazyme Biotech Co.,Ltd, China). Relative mRNA levels were normalized to GAPDH. Primer sequences were listed in **Table S7**.

**1.14 Cell cycle detection assay**

Following drug treatment, cells were harvested by centrifugation at 300 × g for 5 min, and the supernatant was carefully removed. The cell pellet was resuspended in phosphate-buffered saline (PBS) and re-centrifuged under the same conditions to wash. Subsequently, cells were fixed by slow addition of ice-cold 70% ethanol while vortexing, and stored at 4 °C overnight.

On the following day, fixed cells were pelleted by centrifugation and washed once with PBS. For combined phospho-histone H3 (PHH3) and propidium iodide (PI) staining, cells were first incubated with primary anti-PHH3 antibody at room temperature for 1 hour with gentle agitation. Following three washes with PBS, cells were incubated with Alexa Fluor® 488-conjugated anti-mouse IgG secondary antibody (dilution 1:200) in the dark at 4 °C for 1 hour. After three additional PBS washes, cells were resuspended in staining buffer containing 0.05% Triton X-100, 50 μg/mL PI, and 100 μg/mL RNase A, and incubated at 37 °C for 30 minutes in the dark. Finally, cells were washed three times with PBS before analysis using a flow cytometer.

For PI single staining, an identical procedure was followed, omitting the PHH3 antibody and secondary antibody incubations. Instead, cells were directly subjected to permeabilization and staining with PI/RNase solution under the same conditions as described above.

**1.15 Immunofluorescence assay**

Immunofluorescence analysis was performed as previously published^4^. All images were captured using a confocal laser scanning microscope (Nikon AX/AX R with NSPARC, Japan). The primary antibodies used are listed in **Table S2.** For GBM cell lines, secondary antibodies were anti-rabbit IgH (H+L) (Alexa Fluor 647 Conjugate) (RRID：AB_10693544) and anti-mouse IgH (H+L) (Alexa Fluor 488 Conjugate) (RRID: AB_10694704). For GBM patient tissue, secondary antibodies were anti-rabbit IgH (H+L) (Alexa Fluor 488 Conjugate) (RRID：AB_1904025) and anti-mouse IgH (H+L) (Alexa Fluor 488 Conjugate) (RRID: AB_1904023).

**1.16 Co-Immunoprecipitation assay**

U87-MG and U251-MG cells were treated with Z5 for 15 min, followed by lysis in RIPA buffer containing protease and phosphatase inhibitors. Lysates were incubated on ice for 15 min and cleared by centrifugation (12,000 × g, 10 min, 4 °C). An aliquot of each lysate was reserved as whole-cell lysate (WCL) input. The remaining lysate was incubated with anti-EGFR antibody (Santa Cruz Biotechnology) at 4 °C for 1 h with rotation. Pre-washed Protein A/G PLUS-Agarose beads (Santa Cruz Biotechnology) were then added, and the mixture was incubated overnight at 4 °C. The beads were washed three times with ice-cold RIPA buffer, resuspended in 1× SDS loading buffer, and boiled at 95 °C for 10 min to elute bound proteins. Samples were analyzed by SDS-PAGE and immunoblotting with specific antibodies.

**1.17 Clinical Bioinformatics Analysis of WEE1**

Bioinformatic analyses of WEE1 expression and its correlation with patient survival in GBM were conducted using data from the Gliovis database.

**1.18 Subtype Classification of GBM Cell Lines**

To determine the molecular subtypes of the glioblastoma (GBM) cell lines U87-MG and U251-MG, RNA-seq data were obtained from the Cell Model Passports database. For T3359, gene expression microarray data (accession number GSE29750) were retrieved from the NCBI Gene Expression Omnibus (GEO). For D456, RNA-seq data (accession number GSE251878) were also downloaded from GEO.

Single-sample Gene Set Enrichment Analysis (ssGSEA) was performed in R using transcript per million (TPM) values or normalized expression intensities, depending on the data type. The gene signatures defining the proneural, classical, and mesenchymal GBM subtypes were adopted from the landmark study by Verhaak et al^5^.

1. **Supplementary Figures and Legends**


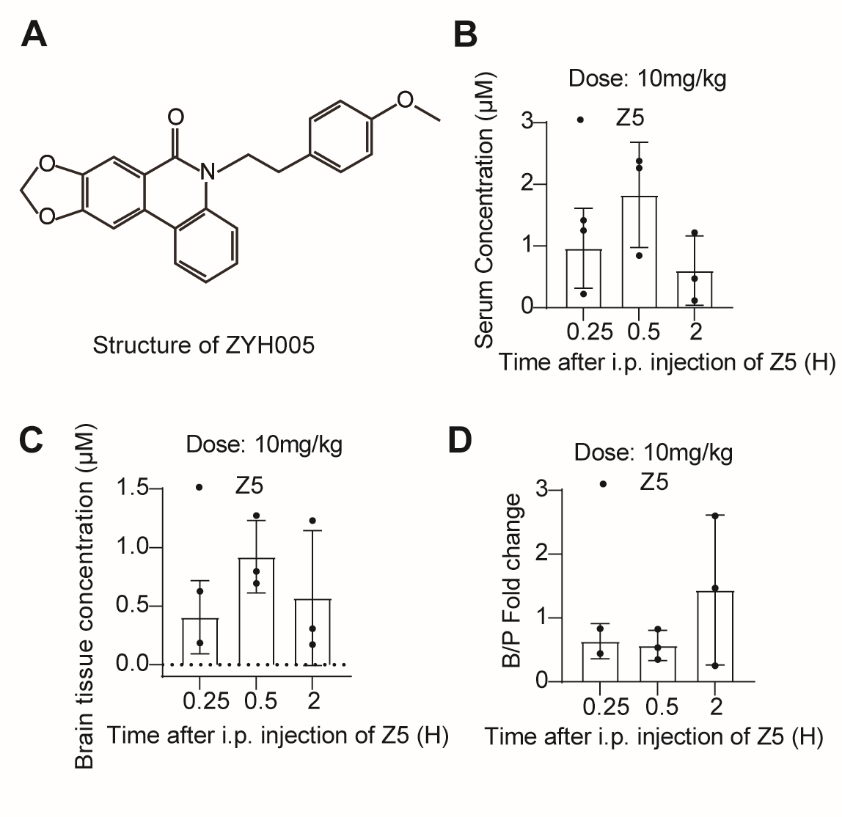


**Figure S1** **Structure and permeability of Z5.** (A) Chemical structure of Z5. (B) Detection of intracranial drug concentrations at different time points after intraperitoneal injection of Z5 using LC-MS/MS (n=3). (C) Detection of plasma drug concentrations at different time points after intraperitoneal injection of Z5 using LC-MS/MS (n=3). (D) Ratio of intracranial drug concentration to plasma drug concentration at different time points after intraperitoneal injection of Z5 (n=3).


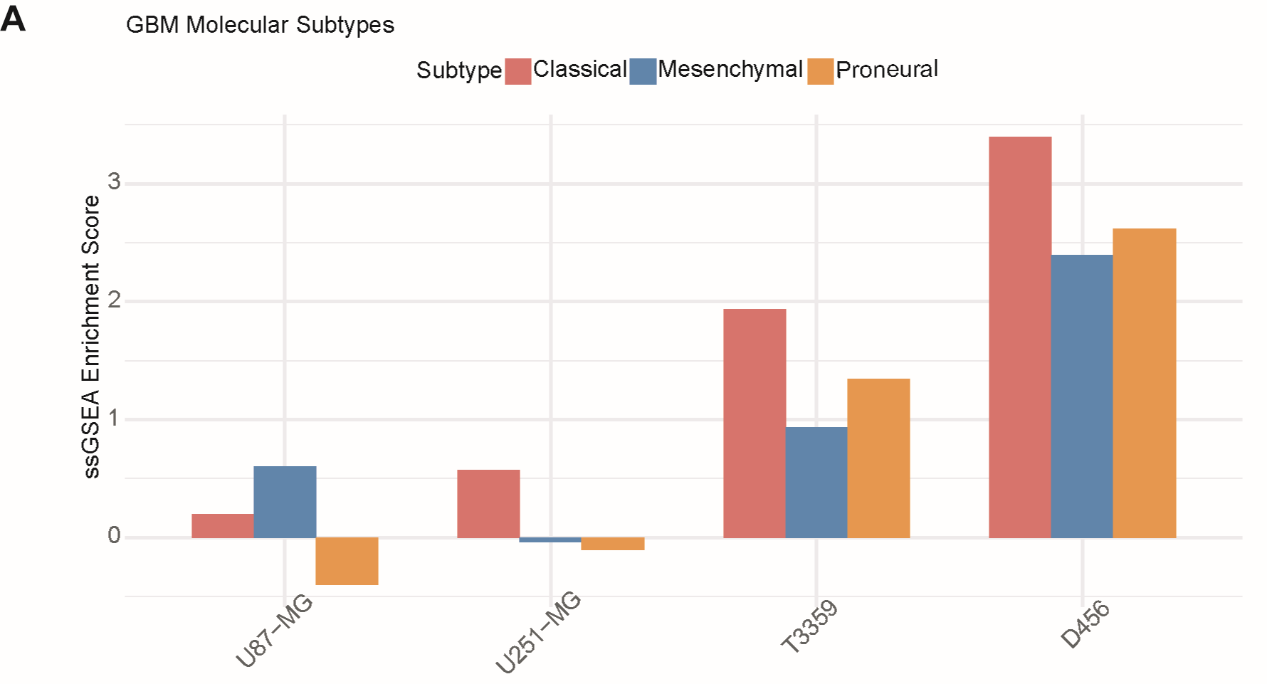


**Figure S2 Molecular Subtype Classification of GBM Cell Lines and Glioma Stem-like Cells.** (A) ssGSEA scores of established GBM cell lines used to distinguish molecular subtypes (proneural, classical, and mesenchymal).


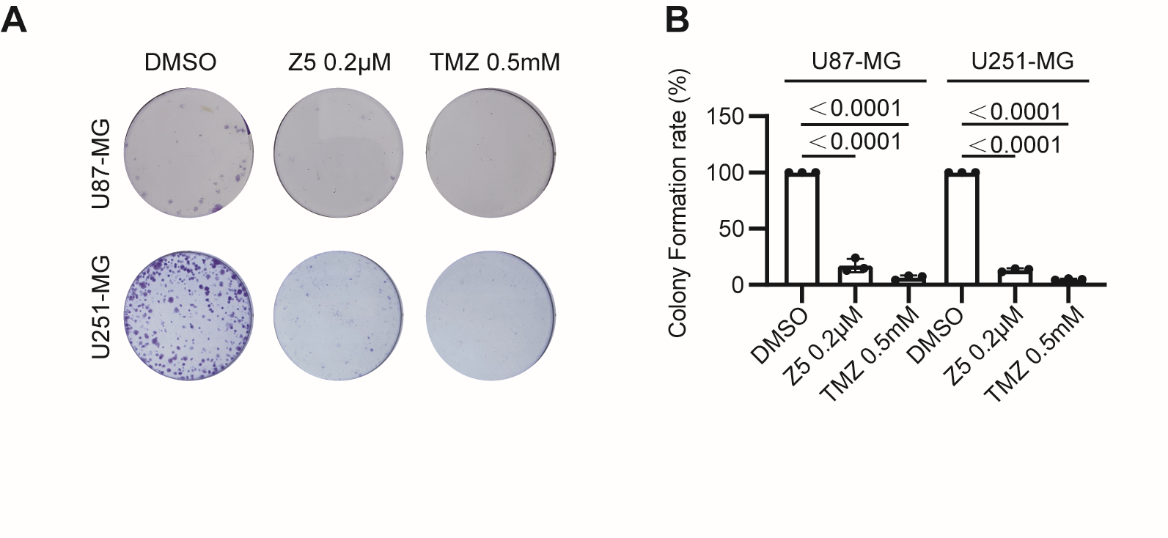


**Figure S3 Z5 and TMZ suppress colony formation in GBM cells.** (A) The inhibitory effect of Z5 or TMZ was evaluated using the colony formation assay. (B) Quantification of colony formation. N=3.


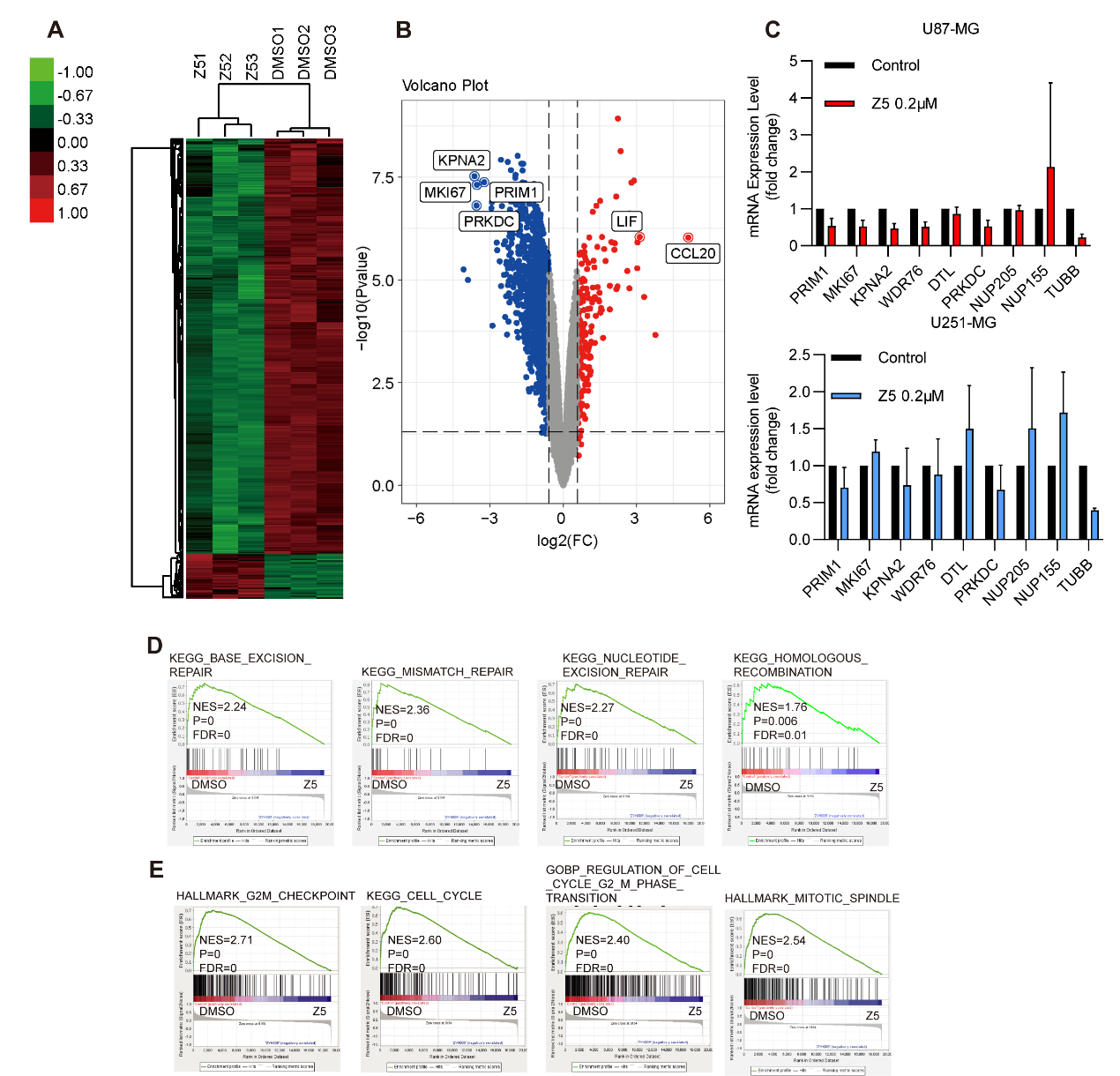


**Figure S4** **Microarray data and GSEA analysis of Z5 treated GBM cells.** (A) Heatmap of microarray data from U87-MG cells treated with DMSO or Z5. (B) Volcano plot showing differentially expressed genes in U87-MG cells after Z5 treatment (|log₂ fold change| > 1, adjusted *p* < 0.05). (C) qPCR validation of selected differentially expressed genes in GBM cell lines. (D) GSEA analysis of base excision repair, mismatch repair, nucleotide excision repair and homologous recombination pathways. (E) GSEA analysis of G2/M checkpoint, cell cycle, cell cycle G2/M phase transition and mitotic spindle pathway.


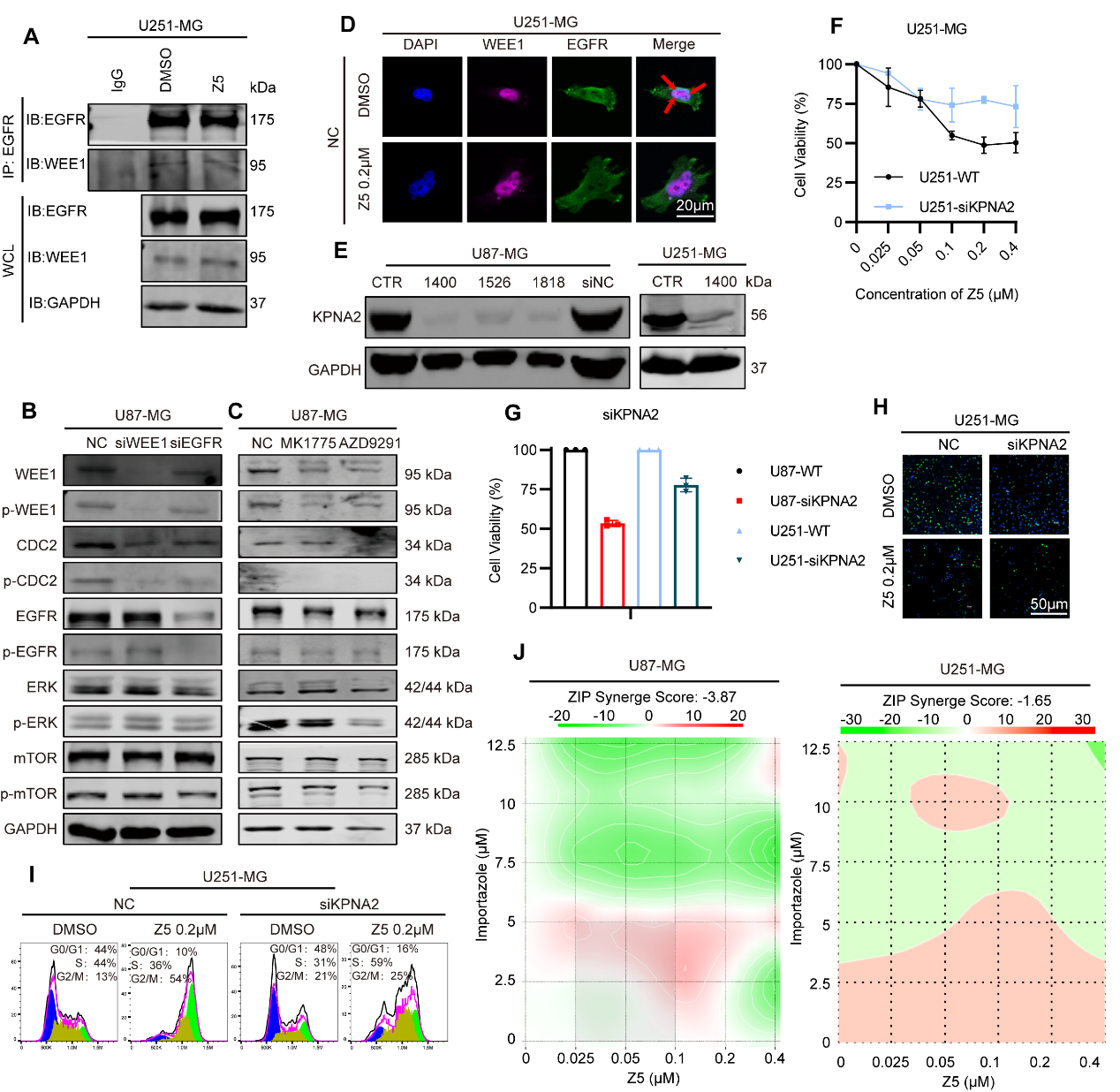


**Figure S5 Z5 disrupt the nuclear EGFR-WEE1 axis.** (A) Co-IP analysis of EGFR–WEE1 interaction in U251-MG cells after 15 min DMSO or Z5 treatment in U251-MG cells. (B-C) Immunoblot analysis of WEE1, p-WEE1, CDC2, p-CDC2, EGFR, p-EGFR, ERK, p-ERK, mTOR, and p-mTOR in U87-MG cells following WEE1 or EGFR knockdown, or treatment with MK1775 (4 μM) and AZD9291 (8 μM). (D)Immunofluorescence (IF) staining was used to analyze the localization of EGFR and WEE1 in U251-MG cells. (E) Efficiency of KPNA2 silencing by siRNA transfection was assessed via immunoblot. (F) Growth curves of NC and siKPNA2-1400 U251-MG cells after 48 h Z5 treatment. n=3. (G) Effect of siKPNA2 transfection on cell viability in GBM cell lines. (H) Anti-proliferative effect of Z5 in NC and si-KPNA2 GBM cells was evaluated by EdU staining. (I) Cell cycle distribution in NC and si-KPNA2 U251-MG cells was analyzed by flow cytometry after 24 h Z5 treatment. ​​(J)ZIP synergy score analysis of the combinatorial effect between Z5 and the KPNB1 inhibitor importazole​​.


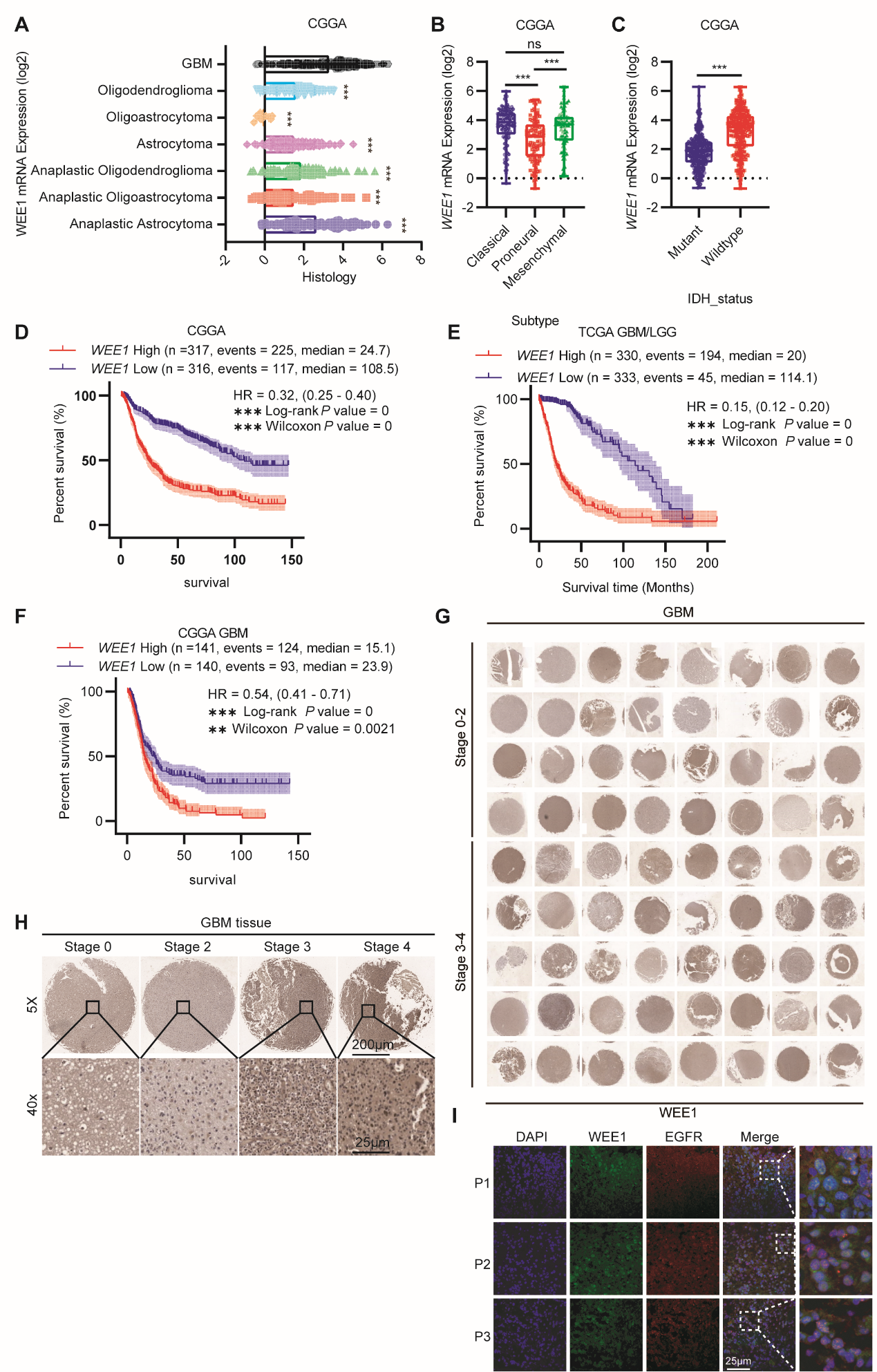


**Figure S6 WEE1 expression correlates with poor survival and EGFR levels.** (A) WEE1 expression across glioma subtypes in the CGGA cohort. (B) WEE1 expression across GBM subtypes in the CGGA database. (C) WEE1 expression in IDH-mutant and IDH-wildtype GBM. Survival analysis based on WEE1 expression in (D) glioma patients (CGGA), (E) GBM/LGG patients (TCGA), and (F) GBM patients (CGGA). (G) Tissue microarray (TMA) of GBM samples. (H) Representative images of WEE1 immunohistochemistry on the TMA are shown (original magnification: upper: 5x; lower: 40x). (I) IF staining images of tumor tissues from GBM patients.


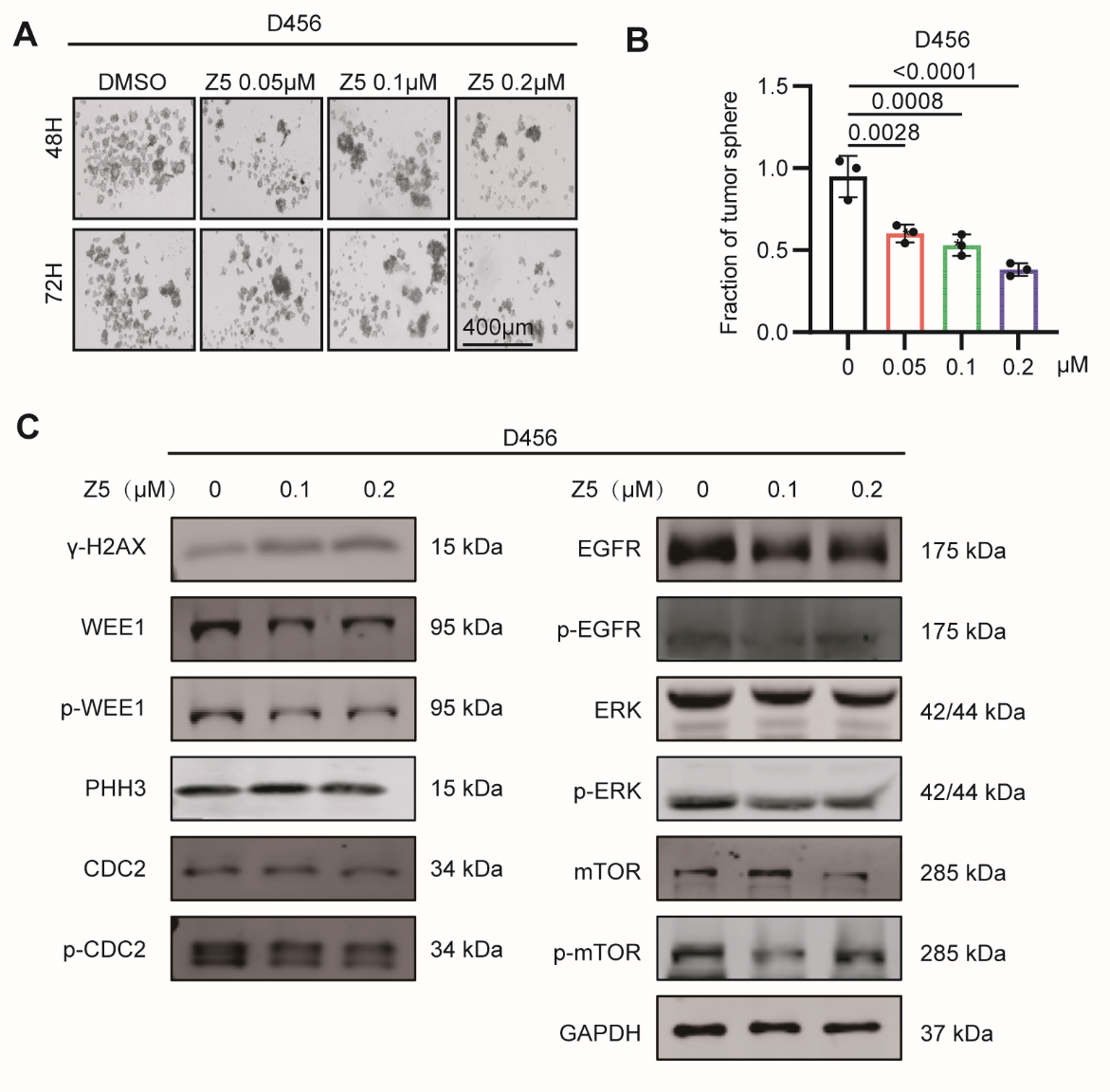


**Figure S7 Z5 suppresses the proliferation of D456.** **​**(A) Representative tumor sphere formation assay in D456 cells treated with different concentrations of Z5 for 48 h. Scale bar = 400 μm. (B) Quantitative analysis of tumor sphere fragmentation from (A). The data are presented as the means ± SDs, n=3, compared with the “0” group. (C) Expression of γ-H2AX, WEE1, p-WEE1, PHH3, CDC2, p-CDC2, EGFR, p-EGFR, ERK, p-ERK, mTOR, and p-mTOR in D456 cells with DMSO or Z5 treatment.


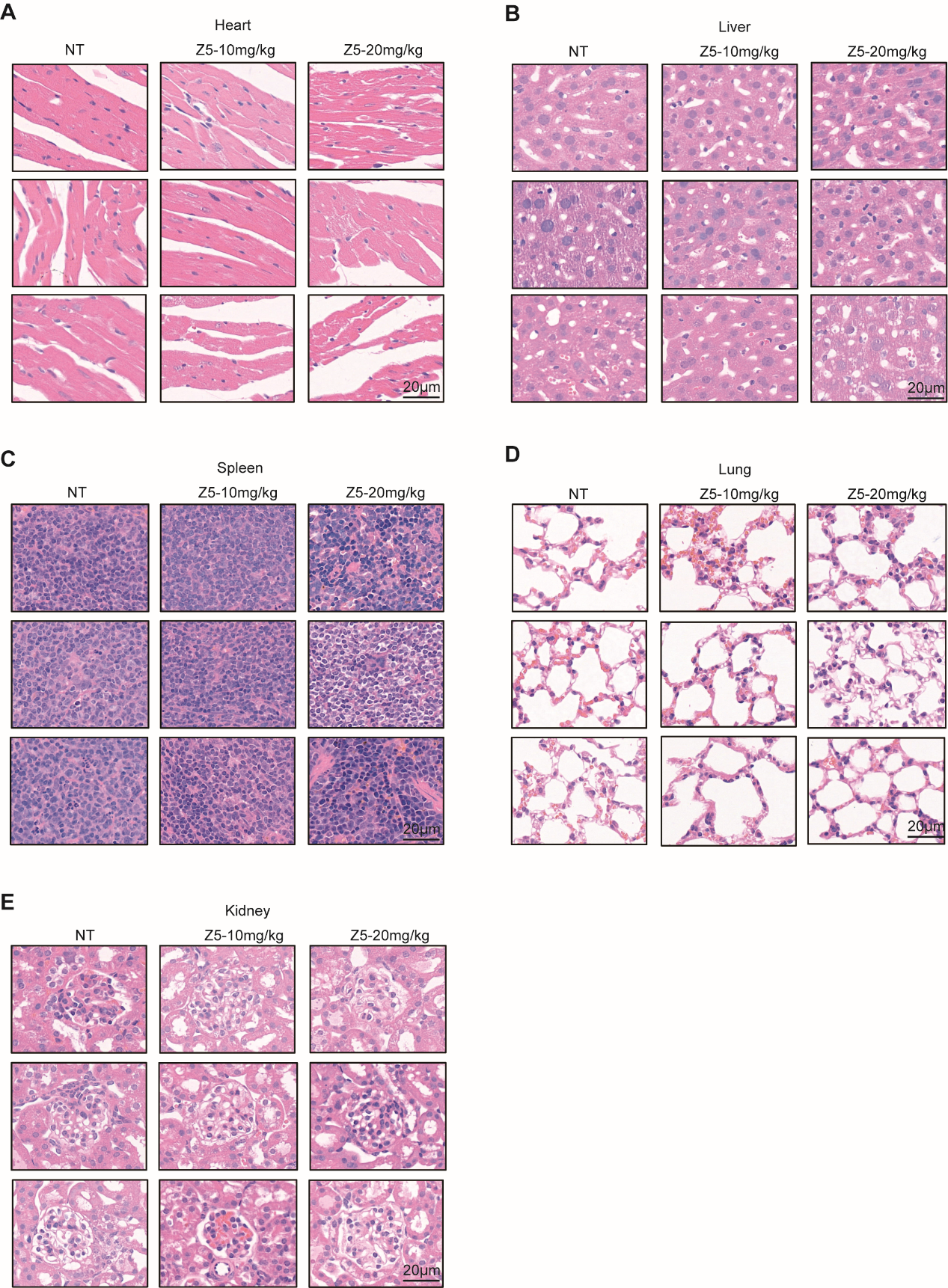


**Figure S8** **Histological Evaluation of Organ Toxicity by Z5 Using H&E Staining.** (A) H&E-stained sections of the heart. (B) H&E-stained sections of the liver. (C) H&E-stained sections of the spleen. (D) H&E-stained sections of the lung. (E) H&E-stained sections of the kidney.


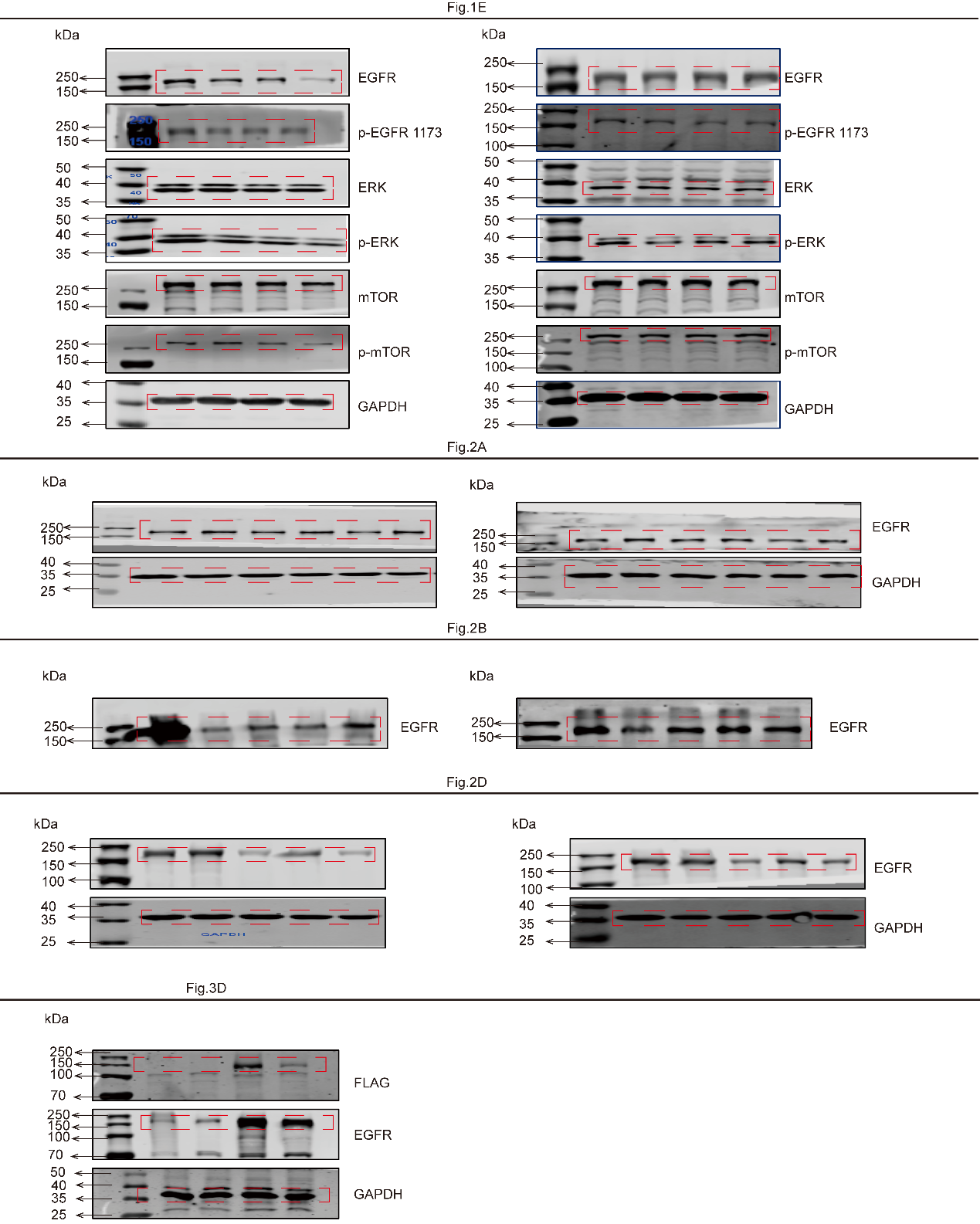


**Figure S9** Complete membrane images of immunoblotting results shown in Fig. 1E, 2A, 2B, 2D and 3D.


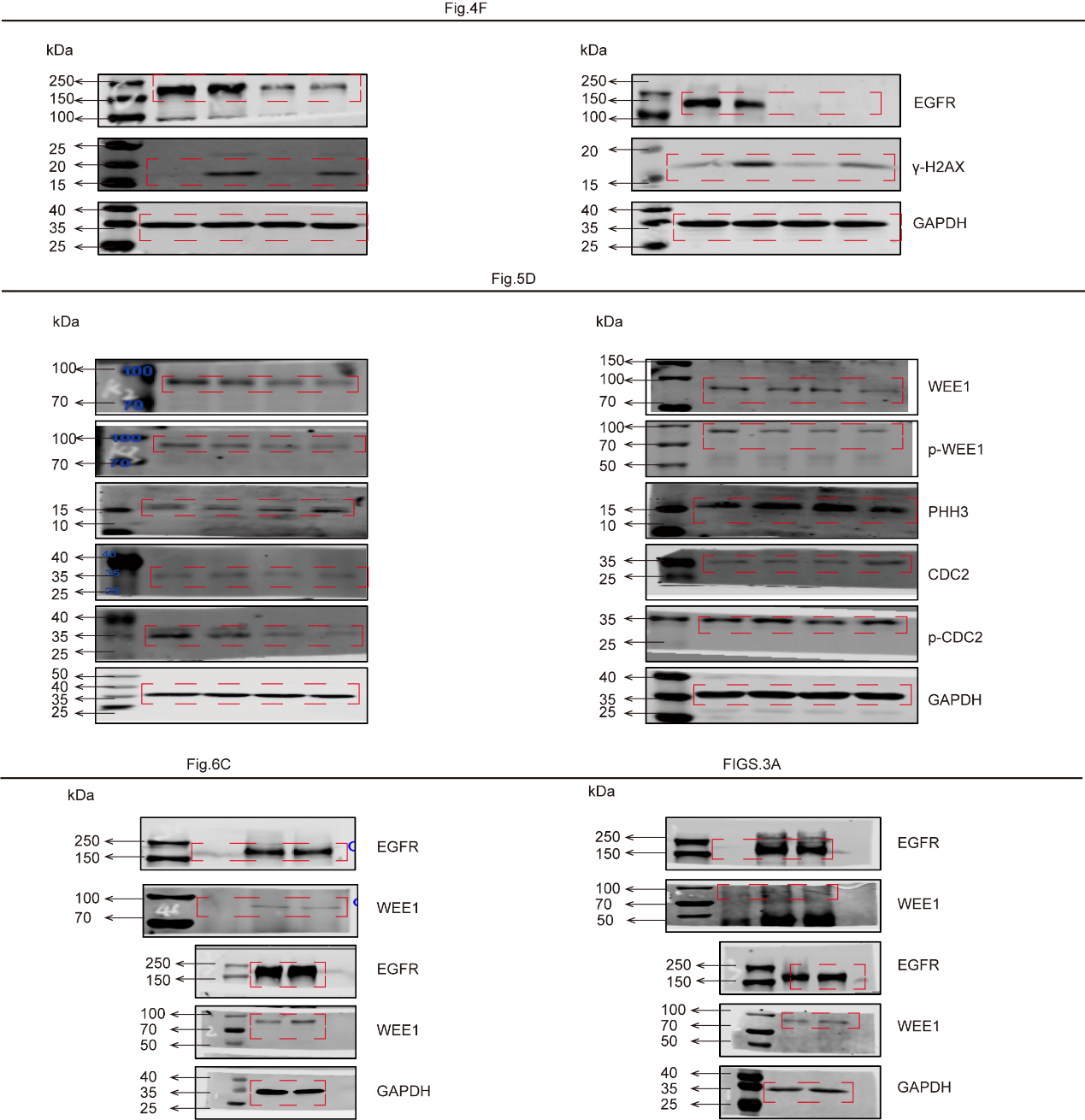


**Figure S10** Complete membrane images of immunoblotting results shown in Fig. 4E, 5D, 6C and FigS.3A.


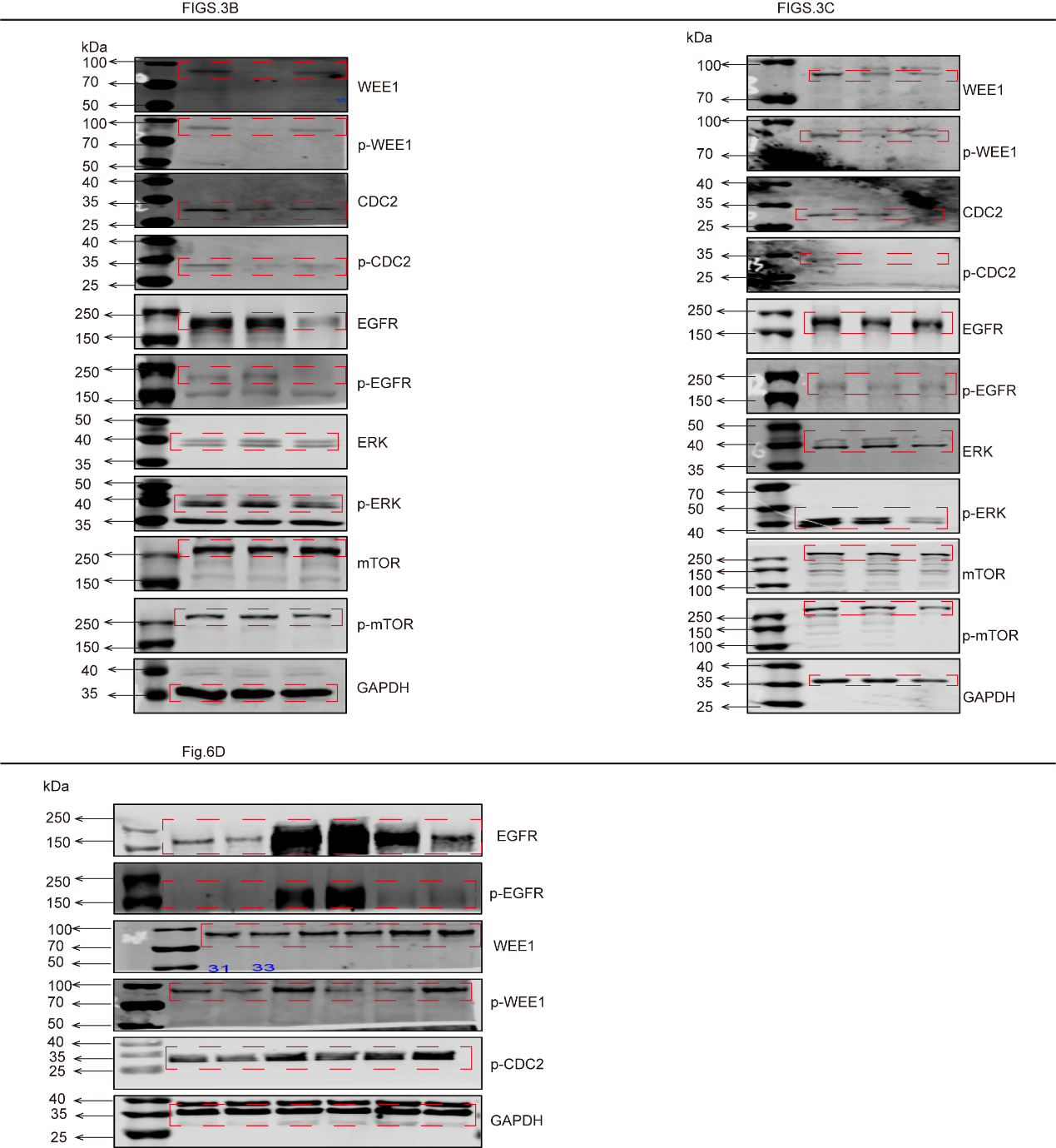


**Figure S11** Complete membrane images of immunoblotting results shown in Fig.6D and FigS.3B, 3C.


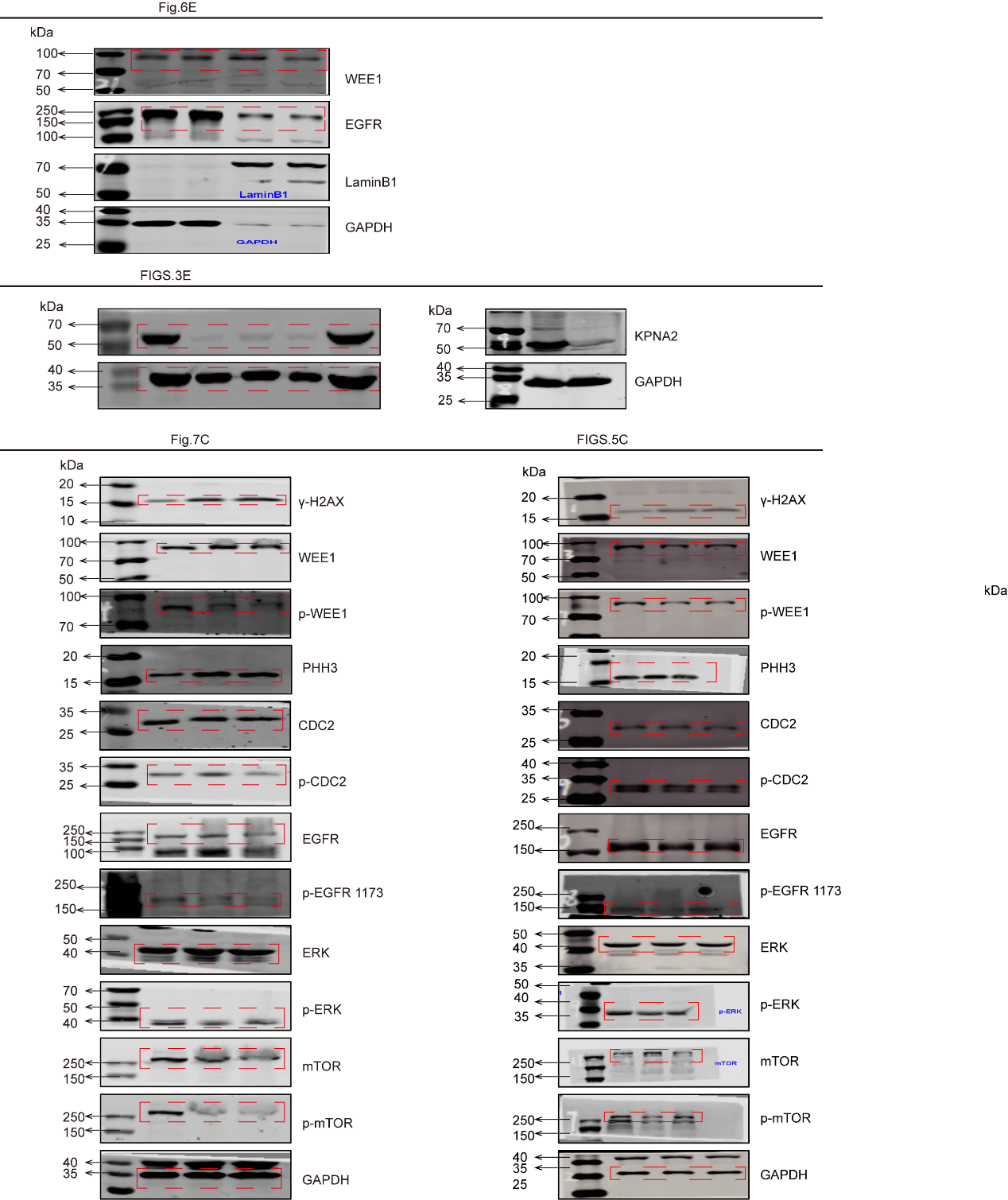


**Figure S12** Complete membrane images of immunoblotting results shown in Fig. 6E, 7C and FigS. 3E, 5C.


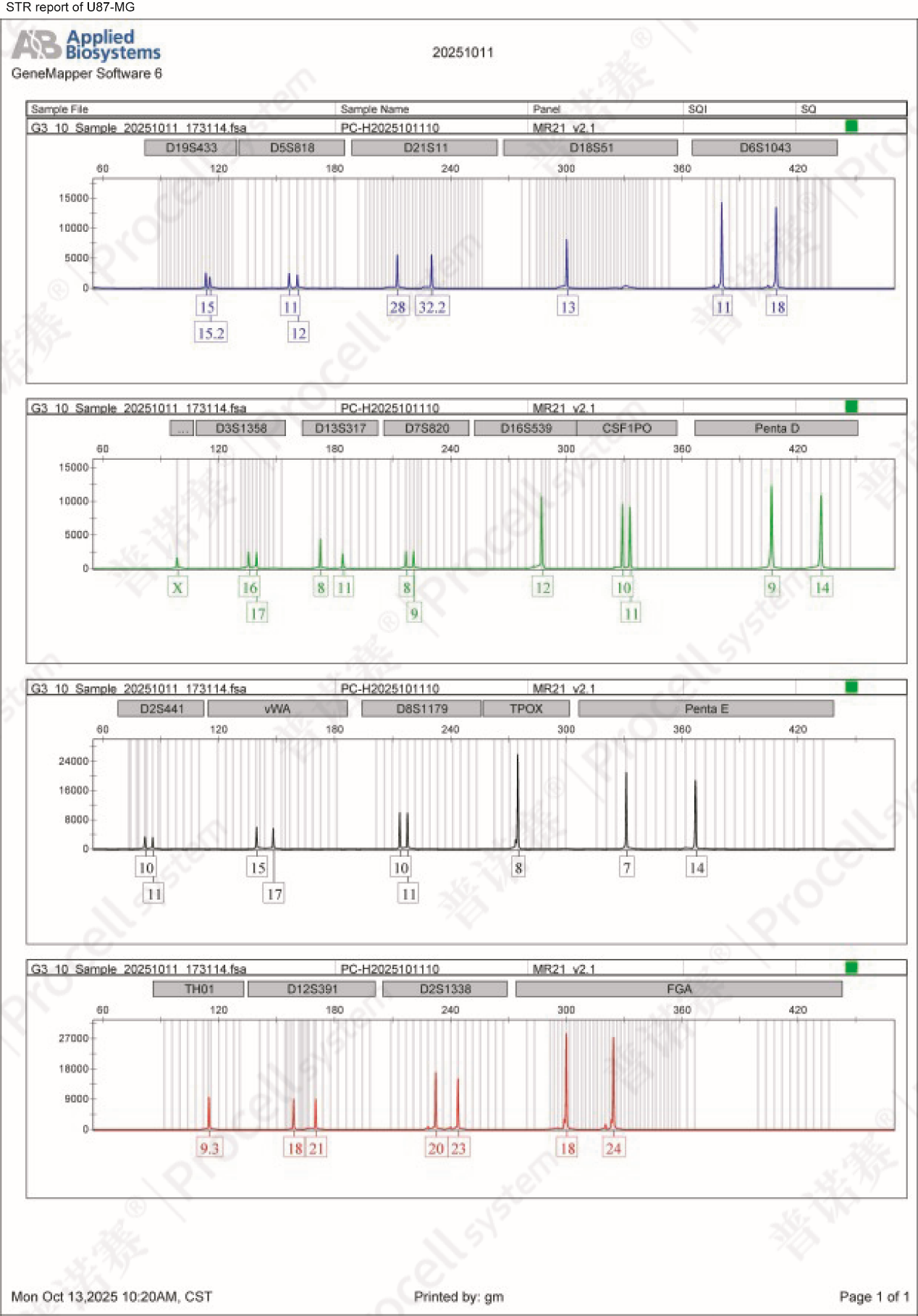


**Figure S13** Results of the latest STR identification for U87-MG cell line.


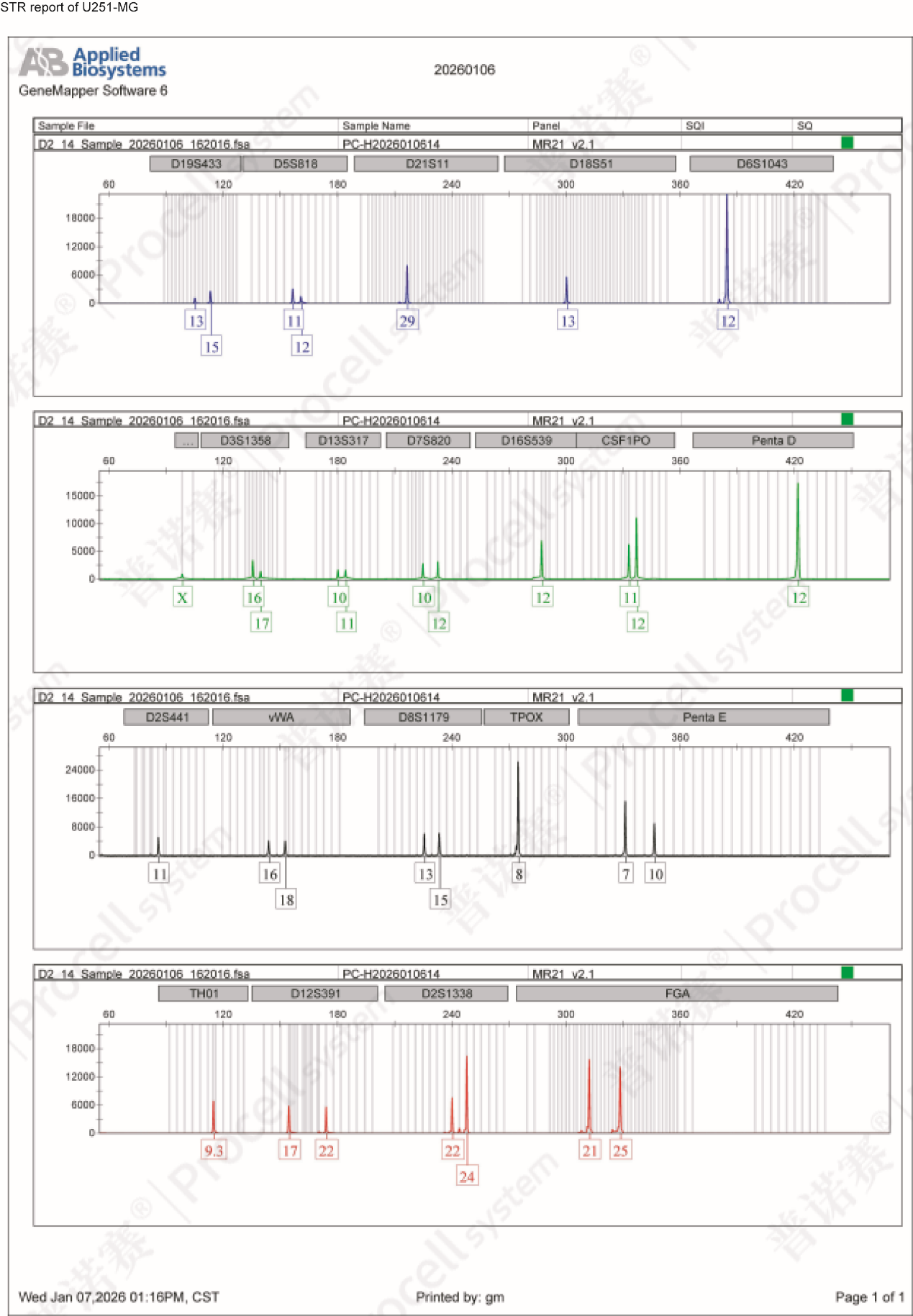


**Figure S14** Results of the latest STR identification for U251-MG cell line.


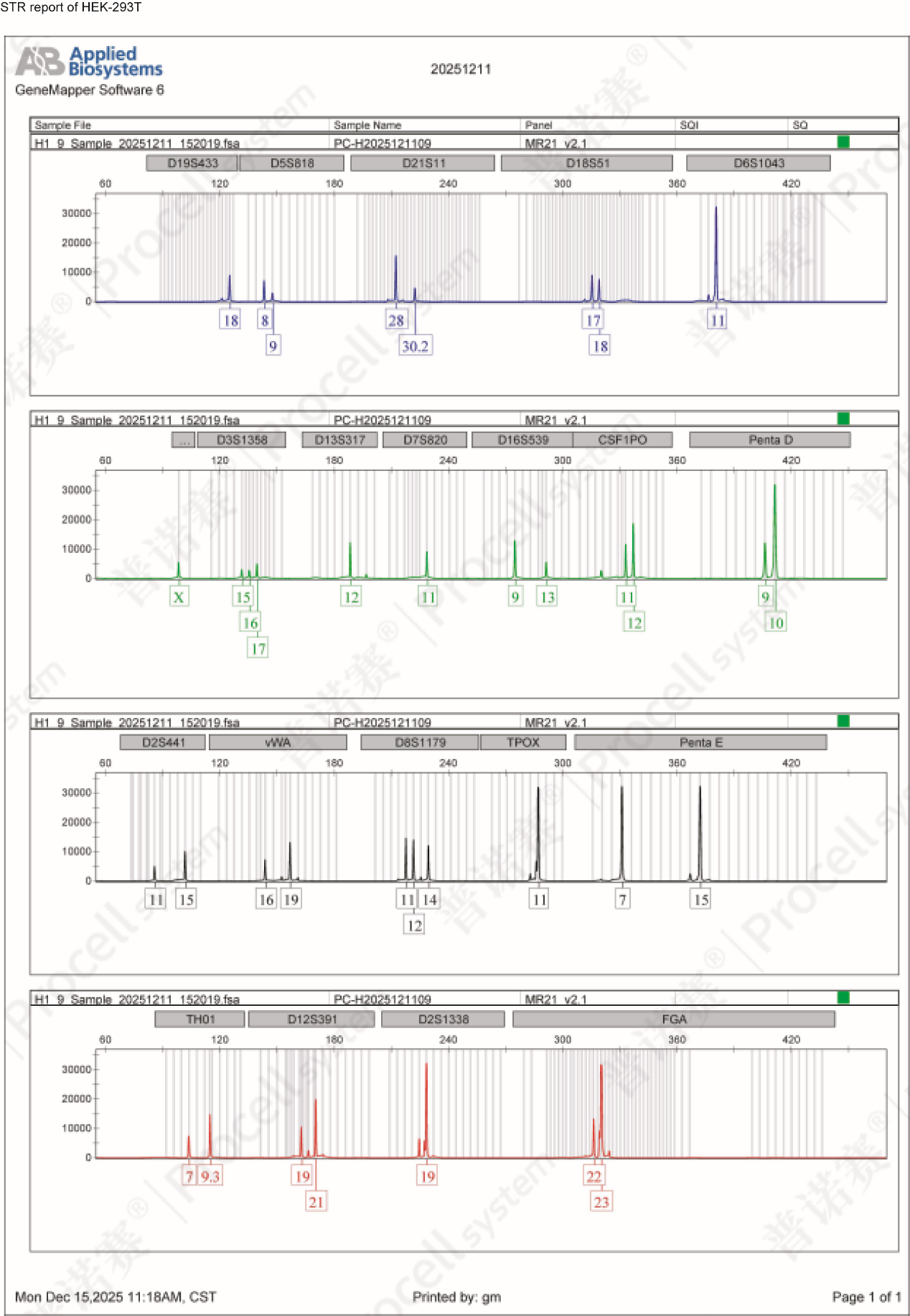


**Figure S15** Results of the latest STR identification for HEK-293T cell line.

1. **Supplementary Tables and Legends**

**Table S1** Differentially Expressed Genes (DEGs) in U87-MG Cell Microarray Data.

Microarray analysis was performed to profile gene expression changes. DEGs were defined by the criteria: |log₂(fold change)| ≥ 1 and false discovery rate (FDR) ≤ 0.05.

Column definitions:

AccID: Official gene symbol of the differentially expressed gene.

log2FC: Log₂-transformed fold change (ZYH005 treatment vs. DMSO control).

Pvalue: Raw two-tailed p-value from the statistical test.

FDR: Adjusted p-value corrected for multiple testing using the False Discovery Rate method.

TU4, TU5, TU6: Normalized expression intensity values (log₂ scale) for individual biological replicates in the ZYH005 treatment group.

TU1, TU2, TU3: Normalized expression intensity values (log₂ scale) for individual biological replicates in the DMSO control group.

Description: Full gene description and transcript information.

Chromosome: Chromosomal location of the gene.

Strand: Transcriptional strand orientation (+ or -).

Start: Genomic start coordinate of the gene.

End: Genomic end coordinate of the gene.

Type: Biotype of the transcript.

**Table S2** Primary antibodies used in the study.

The table summarizes the details of all primary antibodies employed for protein detection and analysis. The columns are defined as follows:

Antigen: The name of the antibody.

Mod-Site: The specific post-translational modification site  recognized by the antibody.

Supplier: The name of the manufacturer.

Product No.: The unique catalog number assigned by the supplier.

Species: The host species in which the antibody was raised (e.g., R-Rabbit, M-Mouse).

**Table S3** siRNA sequences used in the study.

The table lists the sense and antisense strand sequences of siRNAs targeting human EGFR and KPNA2, as well as a non-targeting negative control. All sequences are presented in the 5' to 3' direction.

Column definitions:

Species: Organism source (Homo sapiens).

Symbol: Target gene name followed by a specific identifier for the siRNA sequence (e.g., EGFR-2482). Pairs of rows sharing the same symbol represent the sense and antisense strands of the same siRNA duplex.

The targeting sequence (5’-3’): Oligonucleotide sequence of the siRNA strand.

**Table S4** Plasmid sequences used in the study.

The table provides the sequences of the plasmid used in this study.

**Table S5** Different peptides identified by Lip-MS.

The table lists peptides showing significant abundance changes between the ZYH005 treatment group and the DMSO control group. Selection criteria: |log₂(fold change)| ≥ 2 and adjusted p-value (Padj) < 0.01.

Column definitions:

Protein_Peptide: Unique identifier combining the protein accession and peptide sequence.

Peptide_range: Amino acid position range of the peptide within the parent protein.

PG.Protein Accessions: UniProt accession number of the parent protein.

PG.Genes: Official gene symbol.

PG.Protein Descriptions: Full name of the target protein.

PEP.Stripped Sequence: Amino acid sequence of the peptide.

Mean_T: Mean abundance intensity in the ZYH005 treatment group (replicates E1-1 to E1-3).

Mean_C: Mean abundance intensity in the DMSO control group (replicates E2-1 to E2-3).

T_VS_C_log2fc: Log_2_-transformed fold change (Treatment vs. Control).

T_VS_C_pvalue: Raw two-tailed p-value from the statistical test.

T_VS_C-log10_pvalue: Negative log₁₀-transformed p-value (-log₁₀(p)).

T_VS_C_padjust: Adjusted p-value corrected for multiple testing using False Discovery Rate (FDR).

E1-1, E1-2, E1-3: Raw abundance intensities for individual biological replicates in the ZYH005 treatment group.

E2-1, E2-2, E2-3: Raw abundance intensities for individual biological replicates in the DMSO control group.

**Table S6** RAW data of Lip-MS.

This table presents the complete dataset including all detected peptides and their raw abundance values across experimental replicates. Column definitions for protein information (Protein_Peptide, Peptide_range, PG.ProteinAccessions, PG.Genes, PG.ProteinDescriptions, PEP.StrippedSequence) and statistical metrics (Mean_T, Mean_C, T_VS_C_log2fc, T_VS_C_pvalue, T_VS_C_padjust) are identical to those described in Table S5.

Sample grouping specific to this table:

Z5-1, Z5-2, Z5-3: Raw abundance intensities for biological replicates in the ZYH005 treatment group (corresponding to E1-1/2/3 in Table S5).

CTR-1, CTR-2, CTR-3: Raw abundance intensities for biological replicates in the DMSO control group (corresponding to E2-1/2/3 in Table S5).

**Table S7** Primer sequences used in the study.

All primers were designed to target human (Homo sapiens) genes. Sequences are listed in the 5' to 3' direction.

Column definitions:

Species: Organism source of the gene sequence (Human).

Symbol: Official gene symbol corresponding to the target transcript.

Sequence (5'-3'): Oligonucleotide sequence of the forward or reverse primer.

1. **References**

1. S H. Western Blot Analysis. *Nanotoxicity: Methods in Molecular Biology*. 2012;926:87-97.

2. Hu Y, Zhang Z, Ye W, et al. A DNA structure-mediated fluorescent biosensor for apurinic/apyrimidinic endonuclease 1 activity detection with ultra-high sensitivity and selectivity. *Sensors and Actuators B: Chemical*. 2021;330

3. Pai MY, Lomenick B, Hwang H, et al. Drug affinity responsive target stability (DARTS) for small-molecule target identification. *Methods Mol Biol*. 2015;1263:287-98.

4. Im K, Mareninov S, Diaz MFP, Yong WH. An Introduction to Performing Immunofluorescence Staining. *Methods Mol Biol*. 2019;1897:299-311.

5. Verhaak RG, Hoadley KA, Purdom E, et al. Integrated genomic analysis identifies clinically relevant subtypes of glioblastoma characterized by abnormalities in PDGFRA, IDH1, EGFR, and NF1. *Cancer Cell*. Jan 19 2010;17(1):98-110.
